# Aerobic Intermittent Hypoxic Training Is Not Beneficial for Maximal Oxygen Uptake and Performance: A Systematic Review and Meta‐Analysis

**DOI:** 10.1111/sms.70088

**Published:** 2025-06-23

**Authors:** Gianluigi Dorelli, Gaia Giuriato, Giovanni Zamboni, Michael Daini, Mattia Cominacini, Luca Giuseppe Dalle Carbonare, Eivind Wang, Ernesto Crisafulli, Federico Schena, Massimo Venturelli

**Affiliations:** ^1^ Department of Neuroscience, Biomedicine and Movement Sciences University of Verona Verona Italy; ^2^ Department of Engineering for Innovation Medicine University of Verona Verona Italy; ^3^ Faculty of Health and Social Sciences Molde University College Molde Norway; ^4^ Respiratory Medicine Unit, Department of Medicine University of Verona Verona Italy; ^5^ Department of Internal Medicine University of Utah Salt Lake City Utah USA

**Keywords:** aerobic performance, hemoglobin, hypoxic training, intermittent hypoxic training, mitochondria density, muscle capillarization, oxygen cascade, VO_2max_

## Abstract

Although many studies have investigated whether aerobic training in hypoxia (IHT) could bring advantages to maximal oxygen uptake (V̇O_2max_) and sea‐level performance when compared to analogous normoxic training (NT), the literature results are inconsistent. This variability may come from differences in population, training protocols, hypoxic methods, and potential bias. Therefore, a comprehensive meta‐analysis with strict inclusion criteria is needed to assess the effects of aerobic IHT on V̇O_2max_ and performance. This study aims to review previous meta‐analyses and analyze all parallel‐design studies examining the effect of aerobic IHT compared to NT on V̇O_2max_ and sea‐level aerobic performance. Systematic research was conducted following PRISMA guidelines regarding the effects of aerobic IHT on sea‐level V̇O_2max_ and performance outcomes. The analysis accounted for characteristics of the population, training protocol, hypoxic environment, and publication details. A total of 35 studies involving 524 participants were included. The analysis showed that IHT, compared to NT, did not significantly improve V̇O_2max_ (*p* = 0.333), peak power output (*p* = 0.159), and time to exhaustion (*p* = 0.410). Subgroup analyses identified no significant differences based on fitness level (*p* = 0.690) and exercise modality (*p* = 0.900); however, a publication bias was found (*p* = 0.004). These results suggest that, despite some enthusiastic findings in the literature, possibly influenced by publication‐related biases, aerobic IHT does not offer superior improvement in V̇O_2max_ and performance compared with NT. Therefore, adding hypoxia to aerobic exercise does not enhance training adaptations.

## 1 Introduction

Since the 1960s, athletes and researchers have shown interest in altitude training to improve sea‐level performance [1, 2, 3, 4]. Exposure to hypoxia during exercise increases cardiac output and muscular oxygen extraction, amplifying the physiological response [5, 6, 7]. Based on these observations, intermittent hypoxic training (IHT)—in which hypoxia is applied exclusively during exercise—was hypothesized to be superior to normoxic training (NT) in promoting adaptations of maximal oxygen uptake (V̇O_2max_) and aerobic performance [1]. However, after decades of research, while some studies [8, 9, 10, 11, 12, 13, 14, 15] have reported advantages of IHT over NT for V̇O_2max_, maximal workload, and performance, others have found no such benefits [16, 17, 18, 19].

### 1.1 Heterogeneity in the Literature

Contradictions extended beyond individual studies to systematic reviews. Following the seminal works of Levine [2] and Hoppeler et al. [3], which questioned the advantages of IHT on V̇O_2max_ and muscle oxygen extraction compared with NT, Bonetti and Hopkins [20] concluded in 2009 that IHT‐induced changes in V̇O_2max_ and peak power output (PPO) were negligible compared with those observed after NT. Interestingly, a few years later, Montero and Lundby [21] demonstrated that IHT enhanced muscular neoangiogenesis more than NT, potentially improving oxygen extraction. Likewise, Westmacott et al. [22] found that high‐intensity IHT moderately improved V̇O_2max_ more than high‐intensity NT. More recently, Feng et al. [23] highlighted a significant advantage of IHT in athletes, and Yu et al. [24] identified IHT as the most effective altitude training modality for improving sea‐level V̇O_2max_.

### 1.2 A Quantitative Synthesis Must be Strict but Comprehensive

Given the contradictory evidence, a comprehensive meta‐analysis is crucial to synthesize the effect of IHT on V̇O_2max_ and aerobic performance. A critical step in addressing the variability in IHT outcomes is the evaluation of all relevant study features, including population characteristics, training protocols, hypoxic exposure parameters, experimental design, and publication‐related details [25]. It is equally important to identify and, when appropriate, exclude any papers with overlapping participant cohorts or physiologically implausible results.

Moreover, to minimize confounding bias and isolate adaptations related to oxygen transport, strict inclusion criteria should be applied. To avoid bias from carryover and period effects, crossover studies should be excluded [25]. Likewise, studies involving supramaximal intervals and strength training should be omitted, as these interventions may influence performance through adaptations in anaerobic work capacity and muscular efficiency while exerting only limited effects on V̇O_2max_ [26, 27, 28]. Similarly, studies focusing on small‐muscle group training (e.g., knee extensions) should also be excluded due to their distinct demands on oxygen delivery [2, 5].

### 1.3 Aim of This Meta‐Analysis

We hypothesized that IHT offers no advantage over NT in improving V̇O_2max_ and aerobic performance and that specific factors may account for the contrasting evidence in the literature. Accordingly, the primary aim of this study was to systematically synthesize evidence from all parallel‐group trials comparing aerobic IHT and NT, employing strict inclusion criteria and focusing on adaptations in V̇O_2max_ and aerobic performance. To support interpretation, variables related to oxygen delivery, oxygen extraction, and maximal workload were also considered. As a secondary aim, we assessed the methodologies of previous meta‐analyses and conducted moderator analyses to explore potential sources of heterogeneity across studies and reviews.

## 2 Methods

This Systematic Review and Meta‐Analysis was conducted according to the Preferred Reporting Items for Systematic Reviews and Meta‐Analysis (PRISMA) guidelines [25]. Three independent raters (GD, GG, EW) carried out the study selection, data collection, and publication bias assessment. Any divergences were addressed through discussion or consensus with a fourth rater (MV).

### 2.1 Literature Search and Study Selection

A comprehensive literature search was conducted without restriction on the start date, extending until September 2024. The search encompassed the following scientific databases: PubMed, Scopus, Cochrane, and Web of Science. The search terms utilized were: (1) ‘intermittent hypoxic training’ NOT ‘sprint’, and (2) ‘hypoxic’ OR ‘hypoxia’ AND ‘training’ NOT ‘sprint’. Additionally, the reference lists of included articles and relevant reviews were systematically evaluated to identify potentially pertinent studies.

#### 2.1.1 Eligibility Criteria

The authors included studies that met the following criteria: (1) full‐text manuscripts; (2) not reviews; (3) human studies with healthy participants of any sex aged 18–40 years; (4) parallel studies following a whole‐body aerobic IHT with an intervention group living in normoxia and training in hypoxia, a control group living and training in a normoxic environment, including sea‐level pre‐ and post‐training measures. Aerobic IHT was defined as any training that mainly stimulates the aerobic system, in which the intensity was equal to or lower than maximal workload, maximal running speed, or V̇O_2max_, with intervals equal to or longer than 60 s [29].

#### 2.1.2 Exclusion Criteria

Exclusion criteria for studies were as follows: (1) non‐English publications; (2) inclusion of elderly participants, or those with any acute or chronic diseases; (3) exercise modalities that did not involve whole‐body aerobic training; (4) any altitude training paradigm that differed from IHT, or mixed training paradigms; (5) any form of hypoxic training combining IHT with intermittent hypoxic exposure; (6) crossover studies; (7) studies presenting physiologically unexpected results. We scrutinized the studies for physiologically implausible results, observing pre–post values in the NT groups for potential undeclared methodological issues [30, 31, 32]. We excluded papers where a significant increase in maximal workload was accompanied by a significant decrease in V̇O_2max_, or when the large difference between the pre–post changes of these two variables could not be explained by study design and outcomes [30]. Further details are provided in Supporting Information 1.1.

#### 2.1.3 Review of Existing Meta‐Analytical Evidence on Aerobic IHT

The literature was also systematically screened for meta‐analyses that investigated the effects of IHT and NT on adaptations of V̇O_2max_ and aerobic performance. For each review, the following data were extracted: (1) variable of interest, (2) population, (3) number of included studies and total participants, (4) results, (5) number, description, and justification for studies excluded from the current analysis, (6) specific limitations for a synthesis on aerobic IHT.

### 2.2 Data Collection

The list of included articles was organized using a web‐based reference manager, Rayyan (http://rayyan.qcri.org). Before the eligibility screening process, all papers were consolidated into a unified reference list, and duplicate entries were systematically eliminated using an automated tool with a sensitivity threshold set at 90% similarity (Rayyan). The duplicates not detected by the automatic tool were manually excluded. Titles and abstracts of all papers were screened for the inclusion criteria. Full‐text manuscripts were screened in depth for inclusion and exclusion criteria.

Following eligibility and screening, authors extracted data on study characteristics, including sample size, sea‐level pre‐ and post‐intervention variables of interest, as well as data regarding population, training protocol, hypoxia, V̇O_2max_ assessment, and publication. For a detailed description of all the moderators, see Supporting Information 1.2 and 1.3.

#### 2.2.1 Pre‐ and Post‐Training Variables

Sea‐level pre‐ and post‐training variables included were: (1) V̇O_2max_; (2) Maximal workload (PPO, peak running speed, V_peak_, Maximal Aerobic Speed, vV̇O_2max_); (3) Endurance Performance (Time to exhaustion, TTE; Distance Time Trial, DTT); (4) Maximal cardiac output, Q̇_max_; (5) hematological factors, such as plasma volume (PV) expansion, hematocrit (Ht), hemoglobin concentration (Hb), and mass (Hb_mass_); (6) determinants of oxygen extraction, such as muscle capillarization (Capillary Length Density, CLD; capillary‐to‐fiber ratio, C:F) and mitochondrial density (expressed as citrate synthase activity, CS, and percentage of subsarcolemmal mitochondria, SSM).

Since the evaluation of the effects on the oxygen transport requires V̇O_2max_ to be expressed in absolute terms, all values originally reported in relative terms were estimated to absolute values (_abs_V̇O_2max_) using body weight variations, when available (see Supporting Information 1.4).

#### 2.2.2 Data Items and Processing

Variables of interest were expressed as a pre‐ and post‐training mean difference (*md*) ± pre‐post standard deviation (*σ*). When a study included multiple IHT groups, the two corresponding reports showed a halved number of subjects in the NT group. When *md* for each variable was not reported, the post‐training values were subtracted from the pretraining values; when *σ* was not declared, it was computed starting from the variable's mean, standard deviation, and a correlation coefficient of 0.5 [25]. In case of missing data in the text, the values were estimated from figures using specific software (Image J, Townson, MS, USA).

### 2.3 Meta‐Analysis

All the analyses were performed using the *meta* package in R (R Foundation for Statistical Computing, version 4.2.2). The *md* and *σ* data extracted from the studies were transformed into observed standardized mean differences (SMD), utilizing Hedges' g method [33]. Random‐effects (RE) models employing restricted maximum likelihood estimation were used to calculate the study SMD and their 95% confidence intervals (CI).

The pooled effect was also expressed with SMD, and statistical significance was determined with a *p* value threshold < 0.05. Effect sizes were interpreted using the following categories: trivial (SMD < 0.2), small (SMD 0.2–0.6), moderate (SMD 0.6–1.2), large (SMD 1.2–2.0), or very large (SMD 2.0–4.0) [34]. To evaluate heterogeneity among studies, both the *I* ^2^ statistic and Cochrane's Q statistic were employed, where *I* ^2^ values of 20%, 50%, and 75% indicated low, moderate, and high heterogeneity, respectively [35].

### 2.4 Publication Bias and Sensitivity Analysis

Publication bias was investigated through funnel plots' asymmetry, using both Egger's linear regression method test and the trim‐and‐fill method (only with 10 or more reports) [25, 36, 37]. Studies with standardized residuals exceeding ± 3.0 were classified as outliers [38]. To evaluate the robustness of the overall pooled effect estimate, a leave‐one‐out analysis (LOO) was employed, systematically excluding one study at a time and recalculating the pooled SMD for each outcome variable [25]. Multiple reviewers independently evaluated outliers and overly influential studies for methodological issues (GD, GG, MV, EW).

### 2.5 Subgroup and Meta‐Regression Analysis

Each discrete moderator (Table S1 in Data S2) was analyzed to assess its impact on the overall meta‐analysis results using Cochrane's Q statistics [25]. Additionally, each subgroup was independently evaluated, reporting SMD, CIs, *p* values, and trim‐and‐fill, where applicable.

A meta‐regression analysis, instead, was conducted to explore the influence of all continuous moderators. Data are presented with the regression coefficients (*β*), *p* values, and CIs [25]. In cases where two or more moderators may influence the outcomes, collinearity was assessed using Yates's chi‐squared test (*χ* ^2^) [39]. To avoid the overfitting and low predictive value of the effect, meta‐regression analysis and Cochrane's Q statistics were performed only if the variable contained more than 10 reports [25].

## 3 Results

### 3.1 Literature Research

#### 3.1.1 Limitation of Meta‐Analytical Evidence on Aerobic IHT

The literature included five meta‐analyses that evaluated between 115 and 450 subjects [20, 21, 22, 23, 24]. All the characteristics, results, and methodological considerations of the meta‐analytical evidence are shown in Table 1. The major limitations of the previous meta‐analyses on whole‐body aerobic IHT were: (1) a small number of parallel studies and the inclusion of heterogeneous study designs; (2) inclusion of training modalities beyond aerobic IHT; (3) comparison between small‐muscle mass training and whole‐body training; (4) overlapping cohorts across multiple reports; (5) inclusion of papers with physiologically unexpected results, methodological flaws, or specific populations; (6) limited sample size, which precluded robust subgroup analyses and meta‐regression.

**TABLE 1:** Summary of the previous meta‐analysis that compared IHT and NT adaptations, with population, design, aim, results, and limitations.

Authors (Limit of search) Number of studies	Population, design and results (IHT vs. NT)	Excluded studies in the current analysis and reasons (see original papers for reference)	Limitations in aerobic IHT evidence synthesis
Bonetti and Hopkins, 2009 [20] (April 2007) Studies: 8	Healthy adults (*n*: 115); every AT protocol Trivial V̇O_2max_ benefit (1.1% ± 2.0%) No advantage for PO (0.9% ± 2.4%) No data for subanalysis No clear ΔPO/ΔV̇O_2max_ relationship	Hendriksen and Meeuwsen, 2003: crossover Terrados et al., 1988; Truijens et al., 2003: sprint/strength Roels et al., 2005: not IHT Overall: four studies excluded	20+ parallel studies not included Some studies are not specific to aerobic IHT Magnitude‐based inference limits comparability
Montero and Lundby, 2016 [21] (September 2015) Studies: 18	Healthy adults (*n*: 331); IHT Small benefit for muscle capillarization (0.40 [0.10; 0.70]) Moderate advantage for CLD (0.83 [0.31; 1.36])	Shi et al., 2014; Shi et al., 2013: crossover Terrados et al., 1988; Kon et al., 2014; Kong et al., 2014; Schreuder et al., 2014: sprint/strength Melissa et al., 1997; Terrados et al., 1990: single‐leg Desplanches et al., 1996: high‐altitude natives More than four studies: capillarization not the main outcome Overall: 13 studies excluded	Small‐muscle mass training included Study on high‐altitude natives Some studies are not specific to aerobic IHT
Westmacott et al., 2022 [22] (date not specified—end of 2021) Studies: 9	Healthy adults (*n*: 194); high‐intensity IHT Large benefit on VO_2max_ (1.14 [0.56–1.72]) No relationships among ΔVO_2max_, FiO_2_, duration	Zebrowska et al., 2019: crossover Chapman et al., 1998: retrospective Park and Lim, 2017; Richardson et al., 2016; Truijens et al., 2003: sprint/strength Menz et al., 2016: single‐leg Overall: six studies excluded	20+ parallel studies not included Small‐muscle mass training included Some studies are not specific to aerobic IHT
Feng et al., 2023 [23] (date not specified—end of 2022) Studies: 19	Athletes (*n*: 357); comparison among different AT paradigms Small advantage for V̇O_2max_ (0.36 [0.10; 0.62]) IHT is less beneficial for V̇O_2max_ improvement than Live High Train Low paradigms	Hendriksen and Meeuwsen, 2003: crossover Truijens et al., 2002; Arezzolo et al., 2020; Ambrozy et al., 2020: sprint/strength Ponsot et al., 2006 the same cohort as Dufour et al., 2006 (only one should be considered) Czuba et al., 2019: physiologically unexpected results in NT group Teległów et al., 2022: V̇O_2max_ estimated, not measured Overall: eight studies excluded	Eight parallel studies in athletes not included Duplicate cohorts with no clarity: possible overestimation Two studies with methodological limitation Some studies are not specific to aerobic IHT
Yu et al., 2023 [24] (March 2023) Studies: 19	Healthy adults (*n*: 450); comparison among different AT paradigms IHT beneficial for V̇O_2max_ (results not available—Bayesian meta‐analysis) IHT the most effective AT in the overall population to improve V̇O_2max_	Wang et al., 2011; Wang et al., 2017; Chen et al., 2018: likely share the same cohort as Wang et al., 2010 Kong et al., 2017; Park et al., 2017; Park et al., 2018; Ambrozy et al., 2020: sprint/strength Hein et al., 2021: elderly Roels et al., 2005: not IHT Haufe et al., 2008: physiologically unexpected results in NT group Overall: 10 studies excluded	20+ parallel studies not included Duplicate cohorts likely: possible overestimation. 1 study on elderly Some studies are not specific to aerobic IHT 1 study with methodological limitation

*Note:* Results are expressed in standardized mean difference (SMA) and 95% confidence interval (CI).

Abbreviations: AT, altitude training; CLD, capillary length density; Δ, pre–post difference; FiO_2_, fraction of inspired oxygen; IHT, intermittent hypoxic training; NT, normoxic training; PO, power output; V̇O_2max_, maximal oxygen uptake.

#### 3.1.2 Systematic Research and Report Definition

**FIGURE 1:**
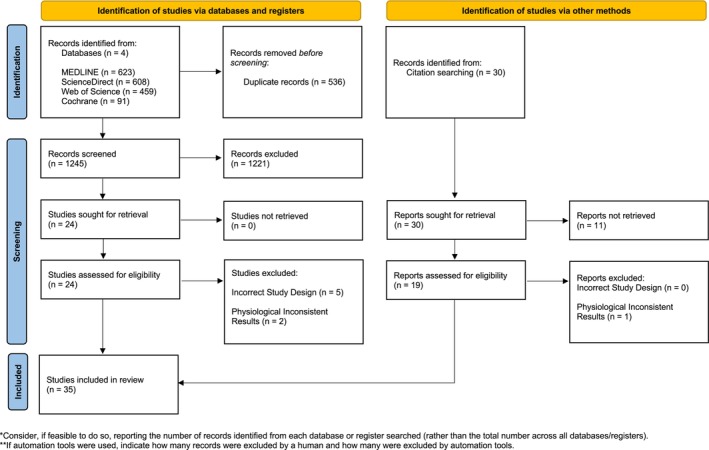
PRISMA flowchart illustrating the systematic review of the literature: A total of 31 reports were identified for the IHT‐NT comparison, derived from 35 individual studies.

A total of 1781 articles from four databases were retrieved, and 35 articles were included, as shown in Figure 1. See Supporting Information 2.1 for the reasons that led to the exclusion of three studies for inconsistent results. Some studies reported or were likely based on the same participant cohort (see Supporting Information 2.2). As a result, 31 reports were considered, each representing a unique cohort. The number of participants in the analysis was 524 (IHT: 276; NT: 248) with a pooled age of 24 ± 4 years (IHT: 23 ± 4; NT: 25 ± 4 years). See Table 2 for the complete description of the studies.

**TABLE 2:** Summary of the studies included in the meta‐analysis.

Study name (Publication data)	Study characteristics	Training description	Findings (IHT/NT): *md* ± δ, Δ%
Desplanches et al., 1993 [40] (France)	CT; Male Sedentary Subjects; FiO_2_: 11.4% – 4800 m (HD) IHT/NT (*n*: 5/5) Age: 22 ± 1/23 ± 1 years Sampling: Mixing Chamber Criteria for maximal test: La_max_ ≥ 8 mmol∙L^−1^; RER ≥ 1.0; V̇O_2_ plateau	Cycling, MIT Weeks: 3; Total Days: 18 1 h (morning) + 1 h (afternoon) at 75% of V̇O_2max_ (N/H) Exclusive Training	V̇O_2max_: 0.25 ± 0.33/0.10 ± 0.57 L∙min^−1^ 6.4%/2.6% C:F: 0.25 ± 0.36/0.45 ± 0.53 13.1%/25.0% CLD: 19 ± 79/108 ± 132 mm∙mm^−3^ 4.2%/22.9% SSM: 0.16 ± 0.39/0.67 ± 0.61% 42.1%/77.9%
Engfred et al., 1994 [41] (Denmark)	CT; Male & Female Sedentary Subjects; FiO_2_: 15.9%; 2500 m (HHC) IHT_rel_/IHT_abs_/NT (*n*: 7/7/7) Age: 26 ± 7/27 ± 5/26 ± 7 years Sampling: Douglas Bag Criteria for maximal test: La_max_ ≥ 8 mmol∙L^−1^; V̇O_2_ plateau	Cycling, MIT Weeks: 5; Total Days: 25 IHT_rel_: 45′ at 70% V̇O_2max_ (H/N) IHT_abs_: 45′ at the same %PPO correspondent to 70% V̇O_2max_ (N) Exclusive Training	V̇O_2max_: 14.7 ± 2.0/11.2 ± 1.8/14.5 ± 2.30 Δ% TTE (at 85% V̇O_2max_): 39 ± 27/43 ± 26/68 ± 25 min 298.5/179.4/441.3%
Emonson et al., 1997 [42] (Australia)	RCT, Double‐Blinded; Male & Female Sedentary Subjects FiO_2_: 15.8%; 2500 m (HHC) IHT/NT (*n*: 9/9) Age: 28 ± 17/30 ± 14 years Sampling: Mixing Chamber Criteria for maximal test: La_max_ ≥ 8 mmol∙L^−1^	Cycling, MIT Weeks: 5; Total Days: 15 45′ at 70% V̇O_2max_ (H/N) Exclusive Training	V̇O_2max_: 6.4 ± 9.1/3.8 ± 10.7 mL∙min^−1^∙kg^−1^ 15.7%/9.0% TTE (at 80% V̇O_2max_) 5.8 ± 3.6/4.7 ± 7.5 min 77.4%/66.9%
Katayama et al., 1999 [43] (Japan)	RCT, Blind Male Sedentary Subjects FiO_2_: 11.8%; 4500 m (HHC) IHT/NT (*n*: 7/7) Age: 21 ± 3.1/21.7 ± 3.9 years Sampling: Douglas Bag Criteria for maximal test: RER ≥ 1.0; V̇O_2_ plateau	Cycling, MIT Weeks: 2; Total Days: 10 30′ at 70% of VO_2max_ (H/N) Exclusive Training	V̇O_2max_: 4.0 ± 5.5/2.9 ± 5.7 mL∙min^−1^∙kg^−1^ 7.1%/5.0%
Bailey et al., 2000 [44] (Wales)	RCT, Male Sedentary Subjects FiO_2_: 16.0%; 2100 m (HD) IHT/NT (*n*: 18/14) Age: 22 ± 3/22 ± 1 years Sampling: Douglas Bag Criteria for maximal test: NS	Cycling; HIT Weeks: 4; Total Days: 12 20′ at 70% HR_max_ (w_1_)—75% HR_max_ (w_2_)—30′ at 80% HR_max_ (w_3_) and 85% HR_max_ (w_4_); HR_max_ considered: H/N Exclusive Training	V̇O_2max_: 0.47 ± 0.55/0.16 ± 0.71 L∙min^−1^ 13.5%/4.0% PPO: 15.0 ± 38.6/22.0 ± 51.9 W 4.6%/7.0%
Geiser et al., 2001 [45] (Switzerland)*	RCT, Male Sedentary Subjects FiO_2_: 12.8%; 3850 m (HD) IHT HIT/NT HIT (*n*: 10/8) Age: 23 ± 2/25 ± 3 years Sampling: Breath‐by‐Breath Criteria for maximal test: La_max_ ≥ 8 mmol∙L^−1^; RER ≥ 1.1; V̇O_2_ plateau; Encouragement	Cycling, HIT Weeks: 6; Total Days: 30 30′ at 85% HR_max_ (H/N) Exclusive Training	V̇O_2max_: 10.4 ± 5.8/8.5 ± 4.2 Δ% Hb (both HIT and MIT): 1.0 ± 7.2/−2.0 ± 11.5 g∙L^−1^ 0.6%/−1.3% CLD: 86 ± 40/−2 ± 127 mm∙mm^−3^ 11.7%/−0.3% SSM: 1.05 ± 1.01/0.19 ± 0.69% 105.0%/13.4%
	IHT MIT/NT MIT (*n*: 8/7) Age: 23 ± 3/29 ± 13 years FiO_2_: 12.8%; 3850 m (HD) Sampling: Breath‐by‐Breath Criteria for maximal test: La_max_ ≥ 8 mmol∙L^−1^; RER ≥ 1.1; V̇O_2_ plateau; Encouragement	Cycling, MIT Weeks: 6; Total Days: 30 30′ at 77% HR_max_ (H/N) Exclusive Training	V̇O_2max_: 5.4 ± 5.3/4.6 ± 8.2 Δ% Hb (both HIT and MIT): 1.0 ± 7.2/−2.0 ± 11.5 g∙L^−1^ 0.6%/−1.3% CLD: 14 ± 102/−74 ± 103 mm∙mm^−3^ 2.2%/−10.1% SSM: 0.74 ± 0.87/−0.13 ± 0.45% 92.5%/−12.9%
Vogt et al., 2001 [46] (France)*	RCT, Male Sedentary Subjects; FiO_2_: 12.9%; 3850 m (HD) IHT HIT/NT HIT (*n*: 7/8) Age: 23 ± 5/25 ± 8 years Sampling: Breath‐by‐Breath Criteria for maximal test: Encouragement	Cycling, HIT Weeks: 6; Total Days: 30 30′ at 85% HR_max_ (H/N) Exclusive Training	V̇O_2max_: 6.7 ± 8.1/4.8 ± 5.5 mL∙min^−1^∙kg^−1^ 13.1%/9.5% PPO: 61.0 ± 33.2/52.0 ± 48.3 W 20.7%/17.7% CLD: 130 ± 137/−30 ± 110 mm∙mm^−3^ 18.5%/−3.8% SSM: 1.21 ± 1.20/0.01 ± 0.75% 130.1%/0.7%
	IHT MIT/NT MIT (*n*: 7/8) Age: 23 ± 5/29 ± 13 years FiO_2_: 12.9%; 3850 m (HD) Sampling: Breath‐by‐Breath Criteria for maximal test: Encouragement	Cycling, MIT Weeks: 6; Total Days: 30 30′ at 77% HR_max_ (H/N) Exclusive Training	V̇O_2max_: 4.9 ± 6.1/4.0 ± 8.5 mL∙min^−1^∙kg^−1^ 10.3%/8.3% PPO: 34 ± 27.87/40 ± 22.45 W 11.4%/13.3% CLD: 14 ± 96/−74 ± 128 mm∙mm^−3^ 2.2%/−10.1% SSM: 0.74 ± 0.72/−0.13 ± 0.48% 100.0%/−12.9%
Masuda et al., 2001 [47] (Japan)	RCT; Male Competitive Team Sports Players Subjects FiO_2_: 15.5%; 2500 m (HHC) IHT/NT (*n*: 7/7) Age: 20 ± 1/19 ± 1 years Sampling: Breath‐by‐Breath Criteria for maximal test: RER ≥ 1.0; V̇O_2_ plateau	Cycling, MIT Weeks: 8; Total Days: 32 First 3 sessions at 60% then 70% of V̇O_2max_ (H/N) Exclusive Training	V̇O_2max_: 5.6 ± 7.0/7.4 ± 15.4 mL∙min^−1^∙kg^−1^ 12.4%/16.5% C:F: 0.40 ± 0.30/0.54 ± 0.45 32.2%/48.3% CS: 27.5 ± 18.9/21.8 ± 24.1 μmol∙min^−1^∙g^−1^prot 32.7%/23.9%
Meeuwsen et al., 2001 [48] (Netherlands)	RCT; Male Competitive Triathletes FiO_2_: 15.9%; 2500 m (HHC) IHT/NT (*n*: 7/7) Age: 28 ± 5/30 ± 5 years Sampling: Breath‐by‐Breath Criteria for maximal test: RER ≥ 1.0	Cycling, MIT Weeks: 1.5; Total Days: 10 2 h at 60%–70% of HRR (N) Part of a Broader Training Program	V̇O_2max_: 162 ± 681/75 ± 688 mL∙min^−1^ 3.4%/1.5% PPO: 26.0 ± 47.5/25.0 ± 51.5 W 6.4%/5.8% Hb: 0.43 ± 0.24/0.34 ± 0.39 mmol∙L^−1^ 4.7%/3.6% Ht: 0.05 ± 0.18/0.05 ± 0.02% 11.6% /11.4%
Messonier et al., 2001 [49] (France)	RCT; Male & Female Sedentary Subjects IHT/NT (*n*: 5/8) Age: 20 ± 1/21 ± 2 years FiO_2_: 13.2%; 3800 m (HD) Sampling: Breath‐by‐Breath Criteria for maximal test: La_max_ ≥ 8 mmol∙L^−1^; RER ≥ 1.1; V̇O_2_ plateau	Cycling, MIT Weeks: 4; Total Days: 24 2 h at 60%–70% (w_1_)—70%–80% (w_2_)—80% (w_3,4_) of PPO (H/N) Exclusive Training	CLD: 103 ± 74/108 ± 67 mm^−2^ 31.4%/29.4% CS: 6.9 ± 2.5/9.9 ± 7.8 mmol∙min^−1^∙g^−1^ 37.9%/54.4%
Ventura et al., 2003 [50] (Switzerland)	CT; Male & Female Competitive Cyclists FiO_2_: 14.1%; 3200 m (HD) IHT/NT (*n*: 7/5) Age: 25 ± 6/25 ± 5 years Sampling: Breath‐by‐Breath Criteria for maximal test: Encouragement	Cycling, HIT Weeks: 6; Total Days: 18 30′ at HR correspondent to VT_2_ (H/N) Exclusive Training	V̇O_2max_: 0.40 ± 6.58/−0.40 ± 4.24 L∙min^−1^ 0.6%/−0.6% PPO: 3 ± 46.6/−14.0 ± 39.1 W 0.8%/−3.6% Hb: 2.5 ± 9.6/ −2.2 ± 13.1 g∙dL^−1^ 1.6%/−1.4% Ht: 0.0 ± 2.7/−0.9 ± 3.7% 0.0%/−1.9%
Messonnier et al., 2004 [51] (France)	RCT, IHT/NT (*n*: 5/8) Male & Female Sedentary Subjects; FiO_2_: 13.2%; 3800 m (HD) Age: 20 ± 1/21 ± 2 years Sampling: Breath‐by‐Breath Criteria for maximal test: La_max_ ≥ 8 mmol∙L^−1^	Cycling, MIT Weeks: 4; Total Days: 24 2 h at 60%–70%, 70%–80% and 80% of PPO Exclusive Training	V̇O_2max_: 4.9 ± 7.5/3.6 ± 4.7 mL∙min^−1^∙kg^−1^ 12.4%/8.3% TTE (at PPO): 125 ± 65/187 ± 65 s 43.0%/62.5%
Morton and Cable, 2005 [52] (United Kingdom)	CT, Male Untrained Cyclists FiO2: 14.9%; 2750 m (NHC) IHT/NT (*n*: 8/8) Age: 21 ± 1/20 ± 1 years Sampling: Douglas Bag Criteria for maximal test: Encouragement	Cycling, HIT Weeks: 4; Total Days: 12 10 x [1′ at 80% of PPO (N); 2′ rec at 50% of PPO (N)]. Intensity increased by 5% (after six sessions)—a further 5% (after nine sessions) Part of a Broader Training Program	V̇O_2max_: 3.7 ± 8.6/4.3 ± 5.5 mL∙min^−1^∙kg^−1^ 7.2%/8.0% PPO: 48.7 ± 39.8/52.5 ± 47.7 W 15.5%/17.8% Hb: −0.1 ± 1.4/0.2 ± 1.2 g∙dL^−1^ −0.6%/1.4% Ht: −0.1 ± 2.8/0.1 ± 3.2% −0.2%/0.2%
Dufour et al., 2006 [8], Ponsot et al., 2006 [53] & Zoll et al., 2006 [54] (France)	RCT, Not Blinded IHT/NT (*n*: 9/9, Dufour; 8/7, Ponsot; *n*: 9/6, Zoll) Male Competitive Runners Age: 30 ± 6/30 ± 6 years (Dufour) Male Competitive Endurance Athletes Age: 30 ± 7/31 ± 6 years (Ponsot) Male Elite Runners Age: 30 ± 6/31 ± 8 years (Zoll) FiO_2_: 14.5%; 3000 m (HD) Sampling: Breath‐by‐Breath Criteria for maximal test: RER ≥ 1.1; Encouragement	Running, HIT Weeks: 6; Total Days: 12 10′ w/u at 60% of vV̇O_2max_ (H/N) + 2 bouts at vVT_2_ + 5′ rec at 60% VO_2max_ (H/N). Bouts at vVT_2_: 12′ (w_1,4_), 16′ (w_2,5_), 20′ (w_3,6_). After w_4_, vVT_2_ was increased up to the same HR at w1 Part of a Broader Training Program	V̇O_2max_: 3.0 ± 3.8/1.5 ± 3.1 mL∙min^−1^∙kg^−1^ 4.7%/2.5% (Zoll et al.) V̇O_2max_: 3.4 ± 3. 3/0. 8 ± 3.5 mL∙min^−1^∙kg^−1^ 5.4%/1.3% (Ponsot et al.) TTE (at vV̇O_2max_): 224 ± 144 / 58 ± 140 s 41.5%/11.6% CS: 0.1 ± 6.3/−3.2 ± 5.1 IU∙g^−1^∙wet^−1^∙wt^−1^ 0.5%/−16.7% V_peak_: 0.4 ± 0.6/0.4 ± 1.2 km∙h^−1^ 2.0%/2.0% Hb: 0.5 ± 1.31/0.4 ± 1.31 g∙dL^−1^ 3.3%/2.6% Ht: 1.3 ± 3.9/0.9 ± 3.6% 2.9%/2.0%
Roels et al., 2007 [17] (France)	RCT, Not Blinded IHT/NT (*n*: 10/8) Male Competitive Endurance Athletes FiO_2_: 14.4%; 3000 m (HD) Age: 24 ± 1/24 ± 1 years Sampling: Breath‐by‐Breath Criteria for maximal test: RER ≥ 1.1; V̇O_2_ plateau	Cycling, HIT Weeks: 3; Total Days: 15 2 interval training (IT) + 3 continuous training (CT) sessions/w IT: [3 times 15′ w/u + 2′ at PPO (H/N) + 2′ rec] x 2 (6′ of rest in between) CT: 15′ w/u + 60′ at 60% V̇O_2max_ (H/N) + 15′ c/d Exclusive Training	V̇O_2max_: −0.2 ± 2.1/2.9 ± 3.0 mL∙min^−1^∙kg^−1^ −0.3%/5.0% PPO: 22.5 ± 13.2/24.6 ± 9.5 W 6.6%/7.2% CS: −0.7 ± 1.45/1.5 ± 1.8 μmol∙min^−1^∙mg protein^−1^ −3.5%/7.9%
Wang et al., 2010 [55]; Wang et al., 2011 [56]; Wang et al., 2017 [57]; Chen et al., 2018 [58] (Taiwan)	RCT, Not Blinded IHT_rel_/IHT_abs_/NT (*n*: 12/12/12) Male Sedentary Untrained Subjects FiO2: 15%; 2733 m (NHC) Age: 23 ± 1/23 ± 3/21 ± 1 years Sampling: Breath‐by‐Breath Criteria for maximal test: La_max_ ≥ 8 mmol∙L^−1^; RER ≥ 1.1; V̇O_2_ plateau	Cycling, MIT Weeks: 4; Total Days: 20 IHT_rel_: 30′ at 50% HRR (N) IHT_abs_: 30′ at 50% PPO (N) Exclusive Training	V̇O_2max_: 1.7 ± 5.2/7.8 ± 5.0/4.2 ± 10.6 mL∙min ^−1^∙kg^−1^ 4.1%/ 17.9%/9.8% PPO: 26.0 ± 29.6/30.0 ± 32.5/15.0 ± 24.0 W 14.0%/15.2%/7.9% Hb: 0.1 ± 1.4/0.6 ± 3.0/−0.4 ± 2.3 g∙dL^−1^ 1.9%/3.4%/−2.7% Ht: 0.8 ± 2.8/1.0 ± 3.3/−0.8 ± 3.1% 1.7%/2.4%/−1.9%
Debevec et al., 2010 [18] (Slovenia)	RCT, Not Blinded IHT/NT (*n*: 9/9) Male Untrained Cyclists FiO2: 12%; 4500 m (NHC) Age: 20 ± 3/22 ± 4 years Sampling: Douglas Bag Criteria for maximal test: RER ≥ 1.1; V̇O_2_ plateau	Cycling, MIT Weeks: 4; Total Days: 20 5′ w/u at 20% of PPO + 60′ at 50% PPO (H/N) Exclusive Training	V̇O_2max_: 2.2 ± 4.5/6.7 ± 4.3 mL∙min^−1^ ∙kg^−1^ 4.6%/ 14.6% PPO: 8.1 ± 47.3/26.7 ± 33.7 W 2.5%/9.0% Hb: 0.0 ± 9.5/−9.0 ± 11.1 g∙L^−1^ 0.0%/−6.0% Ht: 0.0 ± 3.0/−1.0 ± 3.0% 0.0%/−2.2%
Czuba et al., 2011 [11] (Poland, OA)	RCT, Not Blinded IHT/NT (*n*: 10/10) Male Competitive Cyclists FiO_2_: 12.9%; 3867 m (HD) Age: 22.7 ± 2.7/23.5 ± 3.5 years Sampling: Breath‐by‐Breath Criteria for maximal test: La_max_ ≥ 8 mmol∙L^−1^; V̇O_2_ plateau	Cycling, HIT Weeks: 3; Total Days: 9 15′ w/u at 60%, 70%, 80% of PO_LT_ (N), + 30′ (w_1_), 35′ (w_2_), 40′ (w_3_) at 95% (IHT), or 100% (NT) of PO_LT_ (N) + 15′ c/d at 55% PO_LT_ (N) Part of a Broader Training Program	V̇O_2max_: 183 ± 317/−14 ± 249 mL∙min^−1^ 4.0%/−0.3% PPO: 25.0 ± 31.6/1.0 ± 18.5 W 6.6%/0.3% Ht: 1.90 ± 2.65/1.00 ± 2.65% 4.4%/2.2% Hb: 0.09 ± 1.75/0.00 ± 0.70 mmol∙L^−1^ 1.0%/0.0%
Czuba et al., 2013 [12] (Poland, OA)	RCT, Not Blinded IHT/NT (*n*: 6/6) Male Competitive Team Sports Players FiO_2_: 15.2% (HA)—2500 m (NHC) Age: 22 ± 1.6/22 ± 2.4 years Sampling: Breath‐by‐breath Criteria for maximal test: La_max_ ≥ 8 mmol∙L^−1^; RER ≥ 1.1; V̇O_2_ plateau	Running, HIT Weeks: 3; Total Days: 9 15′ w/u at 60, 70% of vV̇O_2max_ (H/N) + HIIT +5′ c/d at 60% of vV̇O_2max_ (H/N). HIIT: 4 × 4′ (w_1,2_) and 5′ (w_3_) at 90% of vVO_2_ (H/N) with 4′ rec Part of a Broader Training Program	V̇O_2max_: 285 ± 464/67 ± 391 mL∙min^−1^ 6.5%/1.3% PPO: 17.3 ± 53.0/7.4 ± 20.9 W 4.55%/1.74% Hb: 0.2 ± 0.66/0.2 ± 0.6 g∙dL^−1^ 1.3%/1.3% Ht: 0.0 ± 1.8/0.1 ± 1.7% 0.0%/0.2%
Holliss et al., 2014 [19] (United Kingdom)	RCT, Single‐Blinded IHT/NT (*n*: 5/7) Male Competitive Runners FiO_2_: 16%—3000 m (HD) Age: 20 ± 2/19 ± 2 years Sampling: Breath‐by‐Breath Criteria for maximal test: Encouragement	Running, HIT Weeks: 8; Total Days: 16 40′ at PO_LT_ (H/N) Part of a Broader Training Program	V̇O_2max_: −1.0 ± 3.7/3.4 ± 3.1 mL∙min^−1^∙kg^−1^ −1.5%/4.8% TTE (at vV̇O_2max_): −0.3 ± 1.5/0.1 ± 2.1 min −1.1%/ 0.4%
Millet et al., 2014 [16] (Switzerland, OA)	RCT, Not Blinded IHT/NT (*n*: 10/8) Male Competitive Cyclists FiO2: 14.5%; 3000 m (HD) Age: 24.4 ± 0.9/24.2 ± 1.1 years Sampling: Breath‐by‐breath Criteria for maximal test: La_max_ ≥ 8 mmol∙L^−1^; RER ≥ 1.1	Cycling, HIT Weeks: 3; Total Days: 6 60′ at 60% of V̇O_2max_ (H/N) Part of a Broader Training Program	V̇O_2max_: −0.2 ± 2.1/2.9 ± 3.0 mL∙min^−1^∙kg^−1^ −0.3%/5.0% PPO: 22.5 ± 13.2/24.6 ± 9.5 W 6.6%/7.2%
Desplanches et al., 2014 [59] (Switzerland)	RCT, Not Blinded IHT/NT (*n*: 6/6) Male Sedentary Subjects FiO_2_: 12.5%—4000 m (HD) Age: 26 ± 5/29 ± 6 years Sampling: Breath‐by‐Breath Criteria for maximal test: Encouragement	Cycling, MIT Weeks: 6; Total Days: 30 30 min at 65% PPO (H/N) Exclusive Training	V̇O_2max_: 7.5 ± 8.6/4.1 ± 24.0 mL∙min^−1^∙kg^−1^ 17.2%/9.5% PPO: 18.0 ± 53.3/35.5 ± 136.7 W 6.4%/12.0% C:F: 0.27 ± 0.59/−0.01 ± 0.26 15.4%/−0.6% SSM: 1.19 ± 0.58/0.66 ± 0.41% 150.6%/91.7% CLD: 87 ± 127/158 ± 128 mm^‐2^ 14.4%/29.9%
Czuba et al., 2017 [10] (Poland, OA)	RCT, Not Blinded IHT/NT (*n*: 8/7) Male Competitive Swimmers FiO_2_: 15.5%; 2500 m (NHC) Age: 19 ± 1/21 ± 1 years Sampling: Breath‐by‐Breath Criteria for maximal test: La_max_ ≥ 8 mmol∙L^−1^; V̇O_2_ plateau	Cycling, HIT Weeks: 4; Total Days: 8 Circuit training with 4 (w_1–3_) and 5 (w_4_) repetitions. 10′ w/u + 45–55′ main training +10′ c/d. Main Training: upper limb rotator training, then cycle ergometer training: 3′ at 50% V̇O_2max_ (H/N) + 3′ rec + 2′ at 95% V̇O_2max_ (H/N) + 3′ at 50% V̇O_2max_ (H/N) Part of a Broader Training Program	V̇O_2max_: 0.26 ± 0.30/0.17 ± 0.46 L∙min^−1^ 6.1%/4.3% PPO: 26.7 ± 20.6/11.2 ± 36.4 W 7.4%/3.2% Hb: 0.2 ± 0.5/−0.1 ± 0.8 g∙dL^−1^ 1.3%/−0.6% Ht: 0.3 ± 3.6/−0.2 ± 2.2% 0.7%/−0.4%
Czuba et al., 2018 [9] (Poland, OA)	RCT, Not Blinded IHT/NT (*n*: 10/10) Male Competitive Cyclists FiO_2_: 16.3%; 2100 m (NHC) Age: 21 ± 3/22 ± 4 years Sampling: Breath‐by‐breath Criteria for maximal test: La_max_ ≥ 8 mmol∙L^−1^	Cycling, HIT Weeks: 3; Total Days: 9 15′ w/u at 65%–70% of PO_LT_ (H/N) + 30′ (w_1_), 35′ (w_2_), or 40′ (w_3_) at 100% PO_LT_ (H/N) + c/d of 15‐min at 60% PO_LT_ (H/N). Part of a Broader Training Program	V̇O_2max_: 0.16 ± 0.31/0.00 ± 0.22 L∙min^−1^ 3.5%/0.0% PPO: 25.0 ± 33.9/4.0 ± 23.1 W 6.5%/1.1% Hb: −0.1 ± 0.5/0.2 ± 0.8 g∙dL ^−1^ −0.7%/1.3% Ht: 0.2 ± 1.2/0.4 ± 1.8% 0.5%/0.9%
Sanchez & Borrani, 2018 [60] (France)	RCT, Double‐Blinded IHT/NT (*n*: 9/6) Male Competitive Runners FiO_2_: 11% (VA)—5250 m (HD) Age: 29 ± 11/28 ± 7 years Sampling: None Criteria for maximal test: Encouragement	Running, HIT Weeks: 6; Total Days: 18 6 x [5′ at 80%–85% of vV̇O_2max_ (H/N) + 5′ rec] Exclusive Training	TTE (at 95% vV̇O_2max_): 90 ± 107/−10 ± 89 s 26.7%/−2.4% vV̇O_2max_ (estimated): 0.5 ± 0.8/0.0 ± 0.7 km∙h ^−1^ 2.4%/0.1% Hb: −0.1 ± 0.7/−0.2 ± 0.6 g∙dL ^−1^ −0.3%/−1.4% Ht: −0.6 ± 1.9/−1.2 ± 1.1% −1.3%/2.6% PV: 0.9% ± 0.8%/0.7 ± 1.1%
Jung et al., 2020 [15] (South Korea, OA)	RCT, Not Blinded IHT/NT (*n*: 10/10) Male Competitive Runners FiO_2_: 14%; 3000 m (HHC) Age: 26 ± 1/26 ± 2 years Sampling: Breath‐by‐breath Criteria for maximal test: La_max_ ≥ 8 mmol∙L^−1^; V̇O_2_ plateau	Running, HIT Weeks: 6; Total Days: 18 15′ w/u at 50%–70% HR_max_ (N) + 10 x [5′ at 90%–95% HR_max_ (N) + 1′ rec] + 15′ cool‐down at 70%–50% HR_max_ (N) Exclusive Training	V̇O_2max_: 3.8 ± 5.1/1.0 ± 4.3 mL∙min^−1^ kg^−1^ 6.0%/1.5% DTT (3000 m): −27 ± 24/−12 ± 22 s −5.12%/−2.3%
Kim et al., 2021 [61] (South Korea, OA)	CT, Not Blinded IHT/NT (*n*: 10/10) Male Competitive Swimmers FiO_2_: 14.5%; 3000 m (HHC) Age: 24 ± 3/24 ± 3 years Sampling: Breath‐by‐breath Criteria for maximal test: NS	Running and Cycling, HIT Weeks: 6; Total Days: 18 15′ w/u at 50%, 60%, 70% of HR_max_ (H/N) + 30′ at 75% HR_max_ on treadmill + 10 x (2′ at 90% HR_max_ + 1′ rec) on cyclo‐ergometer +15′ c/d at 50%–70% of HR_max_ (H/N) Part of a Broader Training Program	V̇O_2max_: 6.1 ± 5.6/2.4 ± 5.8 mL∙min^−1^∙kg^−1^ 12.0%/4.7% Hb: −0.9 ± 1.3/−0.4 ± 1.5 g∙dL^−1^ −5.6%/2.6% Ht: −2.4 ± 3.3/−0.4 ± 3.6% −5.2%/ −0.9%
Lin et al., 2021 [62] (Taiwan, OA)	RCT, Not Blinded IHT/NT (*n*: 20/20) Male Sedentary Subjects FiO_2_: 15.0%; 2750 m (NHC) Age: 22 ± 3/22 ± 4 years Sampling: Breath‐by‐breath Criteria for maximal test: NS	Cycling, MIT Weeks: 6; Total Days: 30 3′ at 30% PPO (H/N) + 30′ at 60% PPO (H/N) Exclusive Training	V̇O_2max_: 5.4 ± 6.1/2.7 ± 4.9 mL∙min^−1^∙kg^−1^ 16.2%/8.0% PPO: 39.0 ± 22.4/29.0 ± 22.4 W 20.6%/15.5% Hb: 0.1 ± 8.5/−0.1 ± 0.9 g∙dL^−1^ 0.7%/−0.7% Ht: −1.1 ± 2.1/0.0 ± 2.7% −2.7%/0.0%
Park et al., 2022 [14, 63] (South Korea, OA)	RCT, Not Blinded IHT/NT (*n*: 10/10) Female Competitive Runners FiO_2_: 14.5%; 3000 m (HHC) Age: 25 ± 4/25 ± 4 years Sampling: Breath‐by‐Breath Criteria for maximal test: La_max_ ≥ 8 mmol∙L^−1^; RER ≥ 1.1; V̇O_2_ plateau	Running, HIT Weeks: 6; Total Days: 18 20′ w/u at 60% HR_max_ + 10 x (5′ at 90%–95% HR_max_, 1′ rec) + 20′ c/d at 60% HR_max_ Exclusive Training	V̇O_2max_: 7.0 ± 5.4/4.0 ± 4.3 mL∙min^−1^∙kg^−1^ 14.3%/8.1% DTT (3000 m): −35 ± 27/−23 ± 31 s −5.4%/ 3.6% Hb: 0.0 ± 0.7/−0.2 ± 1.0 g∙dL^−1^ 0.0%/−1.4% Ht: 1.5 ± 2.0/−0.3 ± 2.2% 3.4%/−0.7%

Abbreviations: Abs, training targeted on absolute workload; c/d, cool‐down; C:F, capillary‐to‐fiber ratio; CLD, capillary length density; CS, citrate synthase; CT, nonrandomized controlled trial; DTT, distance time trial; FiO_2_, fraction of inspired oxygen; H, relative to hypoxic condition, according to the group; Hb, hemoglobin concentration; HD, hypoxicator; HHC, hypobaric hypoxic chamber; HIT, high‐intensity training; HR_max_, maximum heart rate; Ht, hematocrit; IHT, intermittent hypoxic training group; LT_2_, second lactate threshold; MIT, moderate intensity training; N, relative to normoxic condition; NHC, normobaric hypoxic chamber; NS, not specified in the Article; NT, normoxic training (Control) group; OA, open‐access publication; PO_LT_, power output correspondent to 4 mmol∙L^−1^ of lactate; PPO, peak power output; PV, plasma volume; RCT, randomized controlled trial; Rel, training targeted on HR_max_, or V̇O_2max_; SSM, percentage of volume of subsarcolemmal mitochondria; TTE, time to exhaustion; V̇O_2max_, maximal oxygen uptake; V_peak_, maximal running speed; VT_2_, second ventilatory threshold; vV̇O_2max_, maximal aerobic speed; w, week; w/u, warm‐up.

* studies that likely share the same cohort.

### 3.2 Meta‐Analysis

**FIGURE 2:**
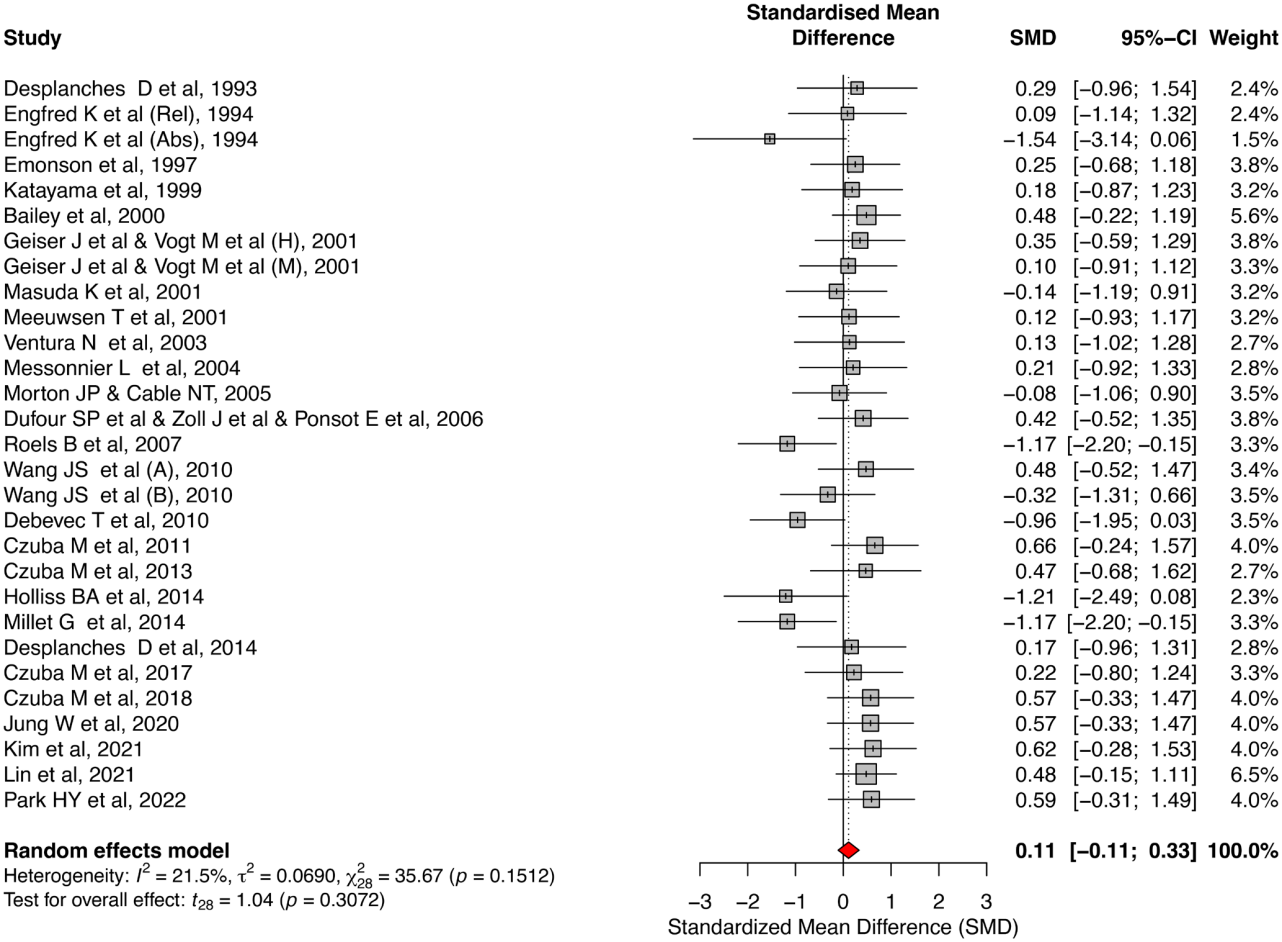
Forest plot from a meta‐analysis depicting the standardized mean differences (SMD) and 95% confidence intervals (CI) for increases in V̇O_2max_ between intermittent hypoxic training (right side) and normoxic training (left side).

Table 3 contains the results of the meta‐analysis. Table S1 contains the sensitivity analysis for each variable. Figure 2 shows the forest plots depicting the pooled effect of IHT versus NT on V̇O_2max_.

**TABLE 3:** Results of the meta‐analysis for the variables related to V̇O_2max_ and aerobic performance.

	Reports (papers)	Participants (female) IHT − NT: overall	SMD (95% CI); *p* value	*I* ^2^	Egger's *p* value	Trim‐and‐Fill SMD; number of studies added	LOO analysis Maximal SMD (influential studies)
Maximal oxygen uptake							
V̇O_2max_	29 (31)	276 (19) − 248 (20): 524 (39)	0.11 [−0.11, 0.33]; *p* = 0.307	22%	**0.005** *	0.11 [−0.11, 0.33]; 0	—
_abs_V̇O_2max_	21 (21)	201 (17) − 171 (16): 421 (33)	0.14 [−0.11, 0.39]; *p* = 0.269	19%	**0.001** *	0.14 [−0.11, 0.39]; 0	0.22 (2)
Aerobic performance							
PPO	16 (16)	165 (1) − 139 (0): 304 (1)	0.14 [−0.06, 0.35]; *p* = 0.159	0%	0.609	0.24 [0.03, 0.45]; 3	—
TTE	5 (4)	37 (7) − 30 (8): 67 (15)	−0.30 [−1.22, 0.61]; *p* = 0.410	36%	NA	NA	—
DTT	2 (3)	40 (20) − 20 (10) − 20 (10)	−0.51 [−1.99; 0.96]; *p* = 0.789	0%	NA	NA	—
Hematologic parameters							
Hb	17 (20)	148 (11) − 128 (10): 276 (21)	0.13 [−0.04, 0.29]; *p* = 0.120	0%	0.245	0.05 [−0.12, 0.22]; 3	0.16 (1)
Ht	15 (17)	138 (11) − 120 (10): 258 (21)	0.08 [−0.14, 0.31]; *p* = 0.462	0%	**0.003** *	−0.15 [−0.40, −0.15]; 6	—
Muscle capillarization							
CLD	5 (5)	23 (1) − 23 (2): 46 (3)	0.15 [−0.79, 1.09]; *p* = 0.684	43%	NA	NA	—
C:F	3 (3)	18 (0) − 18 (0): 36 (0)	−0.06 [−1.4, 1.28]; *p* = 0.864	0%	NA	NA	—
Mitochondrial density							
SSM	4 (4)	29 (0) − 26 (0): 55 (0)	0.45 [−0.87, 1.78]; *p* = 0.355	47%	NA	NA	0.8 (1)
CS	4 (4)	22 (1) − 23 (2): 45 (3)	−0.24 [−1.55, 1.07]; *p* = 0.600	57%	NA	NA	—

Abbreviations: 95% CI, confidence interval; _abs_V̇O_2max_, absolute maximal oxygen uptake; C:F, capillary‐to‐fiber ratio; CLD, capillary length density; CS, citrate synthase; Hb, hemoglobin concentration; Ht, hematocrit; IHT, intermittent hypoxic training group; NT, normoxic training (Control) group; PPO, peak power output; SMD, standardized mean difference; SSM, percentage of volume of subsarcolemmal mitochondria; TTE, time to exhaustion; V̇O_2max_, maximal oxygen uptake.

* *p* < 0.05 for either the pooled effect estimate or Egger's test for publication bias.

#### 3.2.1 Effect of IHT on Maximal Oxygen Uptake and Maximal Workload

IHT did not offer additional benefits over NT for V̇O_2max_ (SMD = 0.11 [−0.11, 0.33]; *p* = 0.307; *I* ^2^ = 22%) or _abs_V̇O_2max_ (SMD = 0.14, [−0.11, 0.39]; *p* = 0.269; *I* ^2^ = 19%). Egger's test showed a publication bias for both V̇O_2max_ (*p* = 0.005) and _abs_V̇O_2max_ (*p* = 0.001) analysis.

The maximal workload was assessed using different metrics across studies: 16 reports used PPO (see Table 2), one employed V_peak_ [8, 53, 54], and another used an indirect estimate of vV̇O_2max_ [60]. IHT did not appear to offer additional benefits over NT for improving PPO (SMD = 0.14, [−0.06, 0.35]; *p* = 0.159; *I* ^2^ = 0%).

#### 3.2.2 Effect of IHT on Endurance Performance

Two studies evaluated DTT using a 3000‐m field test [13, 15]. The remaining five reports assessed TTE with different protocols at intensities above critical power or speed. Two reports [41] examined the ability to sustain 85% of V̇O_2max_, one [51] evaluated the ability to sustain PPO, another [60] assessed the maintenance of 95% of vV̇O_2max_, and a report [42] investigated the ability to sustain 80% of V̇O_2max_ in sedentary subjects. No significant benefits of IHT over NT were observed for TTE (SMD = −0.30 [−1.22, 0.61], *p* = 0.410; *I* ^2^ = 36%) and DTT (SMD = −0.51 [−1.99, 0.96], *p* = 0.142, *I* ^2^ = 0%).

#### 3.2.3 Effect of IHT on Oxygen Transport Components

No parallel studies have investigated the effect of IHT and NT on Q̇_max_ and oxygen extraction using gold‐standard techniques or assessed changes in Hb_mass_ [64]. Only one study reported the percentage change in PV, while 16 and 15 reports (see Table 2) showed the pre–post difference for Hb and Ht, respectively [60]. Only 6 and 7 studies (see Table 2) have capillarization and mitochondrial density measures, respectively.

IHT did not significantly increase Hb concentration (SMD = 0.13 [−0.04, 0.29]; *p* = 0.120; *I* ^2^ = 0%), nor Ht (SMD = 0.08 [−0.14, 0.31]; *p* = 0.462; *I* ^2^ = 0%) compared to NT. Publication bias was found only for Ht analysis (Egger's *p* = 0.003). Neither CLD (SMD = 0.15 [−0.79, 1.09]; *p* = 0.684; *I* ^2^ = 43%) nor C:F (−0.06 [−1.4, 1.28]; *p* = 0.864; *I* ^2^ = 0%) showed a difference between IHT and NT. Similarly, no differences are seen for the increase in SSM (SMD = 0.45, [−0.87, 1.78]; *p* = 0.355; *I* ^2^ = 47%) and CS activity (SMD = −0.24, [−1.55, 1.07]; *p* = 0.600; *I* ^2^ = 57%).

### 3.3 Subgroup Analysis

The detailed results of discrete moderator analysis are shown in Table S2.

#### 3.3.1 Impact of Population Characteristics

The participants' mean age was 24 ± 4 years, and one report included a female‐only cohort [14, 63]. The athletic background and training status did not influence the outcome of IHT when compared to NT for V̇O_2max_ (*Q* = 0.08, *p* = 0.780), _abs_V̇O_2max_ (*Q* = 10.58, *p* = 0.100), and PPO (*Q* = 3.27, *p* = 0.660). However, the country significantly moderated the effect for V̇O_2max_ (*Q* = 27.35, *p* < 0.001) and PPO (*Q* = 43.87, *p* < 0.001), with studies from Poland and South Korea showing larger effect sizes (see Table S2).

#### 3.3.2 Impact of Training and Hypoxia Characteristics

No significant advantage of IHT over NT was observed for _abs_V̇O_2max_ (*Q* = 0.02, *p* = 0.890) and PPO (*Q* = 0.29, *p* = 0.590), regardless of whether the intervention was implemented independently or as part of a comprehensive training plan. Similarly, exercise intensity did not influence the adaptive response to IHT compared to NT for either _abs_V̇O_2max_ (*Q* = 0.00, *p* = 0.980) or PPO (*Q* = 0.52, *p* = 0.470). No differences between running and cycling in the increase of V̇O_2max_ (*Q* = 1.77, *p* = 0.410) and _abs_V̇O_2max_ (*Q* = 4.82, *p* = 0.090) were retrieved when IHT is compared to NT. Exercise modality showed collinearity with the country of publication (*χ* ^2^ = 18.9, *p* = 0.042).

Regarding hypoxia‐related moderators, no significant differences were observed in _abs_V̇O_2max_ (*Q* = 2.55, *p* = 0.280) and PPO (*Q* = 1.07, *p* = 0.590) between IHT and NT, regardless of whether oxygen reduction was achieved via hypoxicator (HD), normobaric hypoxic chamber (NHC), or hypobaric hypoxic chamber (HHC). Additionally, the method used to target exercise intensity in IHT had no significant impact on changes in _abs_V̇O_2max_ (*Q* = 1.15, *p* = 0.760) and PPO (*Q* = 0.62, *p* = 0.890).

#### 3.3.3 Impact of Publication Characteristics

Moderator analyses on _abs_V̇O_2max_ revealed no significant differences for randomization (*Q* = 0.13, *p* = 0.720), blinding (*Q* = 2.39, *p* = 0.120), decade of publication (*Q* = 1.96, *p* = 0.370), or open‐access status (*Q* = 1.87, *p* = 0.170) of the study. However, for V̇O_2max_ and _abs_V̇O_2max_, the largest SMDs were found for studies published after 2010 and for those published in open‐access journals. Similarly, subgroup analyses for PPO indicated significant effects for the decade of publication (*Q* = 5.60, *p* = 0.020) and open‐access publication (*Q* = 7.71, *p* = 0.005), with studies published after 2010 and those available as open‐access reporting larger effect sizes than the other subgroups (see Table S2). These two moderators exhibited collinearity (*χ* ^2^ = 20.1, *p* < 0.001), and open‐access status showed collinearity with country of publication (*χ* ^2^ = 23.1, *p* < 0.001).

### 3.4 Meta‐Regression Analysis

Meta‐regression analyses revealed that none of the continuous moderators significantly influenced the difference between IHT and NT. For pre‐post changes of V̇O_2max_, no significant associations were found for hypoxia level (FiO_2_: *β* = 0.04 [−0.12, 0.19], *p* = 0.659), training duration (total days: *β* = 0.01 [−0.05, 0.06], *p* = 0.843), or training frequency (days/week: *β* = −0.02 [−0.22, 0.18], *p* = 0.815). The detailed results from the meta‐regression analysis are provided in Table S3.

## 4 Discussion

The primary aim of this paper was to determine whether aerobic IHT is superior to NT for enhancing V̇O_2max_ and aerobic performance, through a methodologically rigorous selection of the studies. The secondary aim was to identify factors that may account for the variability observed in the literature by reassessing previous meta‐analytical evidence and performing a moderator analysis on the included studies. In line with earlier reviews, our findings indicate that aerobic IHT is not superior to NT in improving V̇O_2max_ (*p* = 0.307), _abs_V̇O_2max_ (*p* = 0.269), PPO (*p* = 0.159), DTT (*p* = 0.142), and TTE (*p* = 0.410) [2, 3, 4, 20].

### 4.1 Oxygen Delivery and Extraction: No Advantage of IHT Over NT

Given the lack of significant advantage, it is reasonable to infer that both oxygen delivery and extraction adaptation after aerobic IHT are likely comparable to those induced by similar NT [2, 3]. Although the present analysis found no significant difference in Hb (*p* = 0.120) and Ht (*p* = 0.864) changes, none of the included studies reported Hb_mass_ variation. However, previous findings demonstrated that adding hypoxia during high‐intensity interval training for 6 weeks can slightly enhance Hb_mass_ gain (8.4% vs. 3.3%), despite no effect on V̇O_2max_ and PPO [65].

Unfortunately, data regarding Q̇_max_ and oxygen extraction using gold‐standard techniques were also lacking [64]. The current results, however, do not support the notion that IHT is superior to NT in enhancing oxygen extraction, based on findings related to muscle capillarization (CLD: *p* = 0.684; C:F: *p* = 0.864) and mitochondrial density (SSM: *p* = 0.355; CS: *p* = 0.600), aligning with the conclusions of previous authors [2, 3]. Indeed, despite the distinct molecular adaptations in skeletal muscle mitochondria induced by IHT, several studies failed to establish a consistent relationship between these changes and the additional benefits for V̇O_2max_ and PPO [3, 40, 45, 46, 53, 59, 65, 66, 67].

In conclusion, although the literature was poor in relevant data, all these findings suggest that aerobic IHT does not elicit different physiological adaptations compared to NT, and it is unlikely to affect any component of oxygen transport.

#### 4.1.1 Population Characteristics

Our findings showed a marked skewed sex ratio in IHT research (485 males vs. 39 females). The inclusion of only one female cohort [14, 63], and five mixed‐sex studies [41, 42, 49, 50, 51] limited the ability to conclude sex‐specific responses to IHT. Nonetheless, the available data on training status are more comprehensive, and it is noteworthy that IHT did not show significantly different effects compared to NT across sedentary individuals, competitive athletes, and elite athletes (_abs_V̇O_2max_ *p* = 0.530) from various athletic backgrounds (_abs_V̇O_2max_ *p* = 0.100). Although some studies have attributed the lack of response in high‐level athletes to their already reached physiological limit [3, 53], our findings indicate that IHT did not provide additional benefits over NT, also in healthy sedentary untrained subjects, despite their greater potential for improving V̇O_2max_ and performance [3, 68].

Surprisingly, the country in which the experiment was conducted consistently influenced the magnitude of reported effects on V̇O_2max_ (*p* < 0.001) and PPO (*p* < 0.001). Some countries reported more pronounced improvements with IHT compared to NT, suggesting that regional differences in training methodologies, participant characteristics, or assessment protocols may contribute to the observed variability in the literature [9, 10, 11, 12, 13, 14, 15, 61, 63].

#### 4.1.2 Training and Hypoxia Characteristics

Differences in training design did not result in significant advantages of IHT over NT in enhancing V̇O_2max_ and performance, regardless of whether the intervention was implemented independently or as part of a comprehensive training plan (_abs_V̇O_2max_ *p* = 0.890; PPO *p* = 0.590). Variations in exercise intensity also did not significantly influence the adaptive responses to IHT compared to NT (_abs_V̇O_2max_ *p* = 0.980; PPO: *p* = 0.470). Similarly, exercise modality—whether running or cycling—did not yield a statistically significant difference in V̇O_2max_ (*p* = 0.410) and _abs_V̇O_2max_ (*p* = 0.090). It is worth noting that most studies employing running reported favorable trends in V̇O_2max_ [8, 12, 14, 15, 53, 54, 63], and DTT [14, 15, 63] when IHT is compared to NT, despite the nonsignificant pooled outcome for DTT. However, exercise modality correlates with the country (*p* = 0.042), limiting the ability to isolate the independent effect of these two moderators.

Lastly, it should also be noted that neither total training volume (*p* = 0.614) nor training frequency (*p* = 0.596) significantly moderated the adaptation of _abs_V̇O_2max_ in response to IHT compared to NT, suggesting that the cumulative hypoxic exposure associated with IHT is unlikely to produce meaningful benefits for oxygen transport, even if it might result in modest additional increases in Hb_mass_ [65].

No significant effects were also observed for hypoxia‐related characteristics. Changes in _abs_V̇O_2max_ (*p* = 0.280) and PPO (*p* = 0.590) did not differ between IHT and NT, regardless of the method to induce hypoxia (HD, NHC, or HHC). Similarly, they also did not differ across varying levels of hypoxia (FiO_2_: *p* = 0.701). The approach used to match exercise intensity in IHT—whether based on absolute normoxic or relative hypoxic intensity, or expressed as a percentage of V̇O_2max_, maximal heart rate, or workload—also had no significant effect on the changes of _abs_V̇O_2max_ (*Q* = 1.15, *p* = 0.760) or PPO (*Q* = 0.62, *p* = 0.890). Taken together, these findings indicate that variations in training design, exercise modality, hypoxia characteristics, and prescription methods did not confer any advantageous effect of IHT over NT.

#### 4.1.3 Study Design and Publication Characteristics

Subgroup analyses of V̇O_2max_ and _abs_V̇O_2max_ revealed no significant differences between IHT and NT adaptation based on study design or publication characteristics. Randomization (*p* = 0.720), blinding (*p* = 0.120), decade of publication (*p* = 0.370), and open‐access status (*p* = 0.170) did not significantly influence the outcomes, even though larger effect sizes are seen for papers published after 2010 and those that were open‐access. This trend, however, is confirmed by subgroup analyses for PPO, where studies published after 2010 (*p* = 0.020) and those available as open access (*p* = 0.005) reported larger effect sizes compared to earlier or nonopen‐access publications. The reasons for these differences remain unclear. However, these two moderators exhibited collinearity (*p* < 0.001), and open‐access status was also collinear with the country of publication (*p* < 0.001), making it challenging to disentangle the independent contributions of each factor.

### 4.2 Factors Contributing to Literature Heterogeneity

Our findings contrast with those of the meta‐analyses published after 2010 [21, 22, 23, 24]. Acknowledging their different primary aims, many of these meta‐analyses shared recurring methodological choices that are different from the current paper.

To our knowledge, this is the first review to apply rigorous inclusion criteria focused exclusively on whole‐body aerobic IHT. Although supramaximal interval and strength training studies do not primarily target oxygen transport [4, 27, 28], single‐leg exercise protocols might have exaggerated the muscular adaptation with IHT [21], even though an advantage in oxygen extraction remains unclear [2, 66]. Furthermore, some meta‐analyses included studies with methodological flaws and questionable outcomes in NT groups (see Table 1), or they may have overestimated effect sizes due to overlapping participant samples across studies without consistently addressing this issue [23, 24].

However, these minor methodological considerations are insufficient to fully account for the diverging findings observed among earlier reviews [2, 3, 20], our findings, and meta‐analyses published after 2010 [22, 23, 24]. Conducting a systematic review after more than 30 years of heterogeneous research may lead to substantial variability in the included studies. For instance, regarding aerobic IHT, some published meta‐analyses have excluded more than 20 studies (see Table 1) [22, 24]. While this should not be viewed as a limitation, as it reflects each researcher's specific focus, it implies that the outcome could differ substantially.

To date, our meta‐analysis encompasses the largest number of studies on aerobic IHT. Egger's regression test revealed a significant risk of publication bias for both V̇O_2max_ (*p* = 0.005) and _abs_V̇O_2max_ (*p* = 0.001), indicating a possible overrepresentation of studies with significant results in the literature and potentially contributing to the observed contradictions over time [36].

Our analysis, indeed, identified a cluster of studies with larger effect sizes published after 2010 [9, 11, 14, 15, 61, 63]. While we cannot definitively explain this pattern on a physiological basis, these studies might have played a significant role in the different meta‐analytical outcomes. This cluster could have introduced a literature bias for evidence syntheses after 2010, resulting in a disproportionate influence of studies showing a greater advantage of IHT over NT [69, 70].

## 5 Conclusion

In conclusion, current evidence does not support the superiority of aerobic IHT over normoxic training for improving performance and V̇O_2max_ in healthy individuals. Despite the publication bias and a cluster of studies with larger effect sizes, three key observations should be emphasized: (1) Training status and athletic background do not influence the adaptive response to IHT; (2) no specific training characteristics or exercise modalities showed superiority in enhancing IHT outcomes; (3) the type and the dose of hypoxia, do not affect the effectiveness of IHT. IHT has consistently shown no clear advantages; therefore, it should not be recommended as a superior method over NT to improve V̇O_2max_ and aerobic performance.

## 6 Limitations

Although the findings show that IHT is not superior to NT, the analysis had two main limitations. First, the absence of gold‐standard measurements for cardiac output, oxygen extraction, and total blood volume limited the ability to comprehensively evaluate changes in the oxygen transport between IHT and NT. Second, while the moderator analyses did not identify any consistent influence of population characteristics, training parameters, hypoxic conditions, testing procedures, or study design to fully account for the variability in the IHT literature, a cluster of studies reporting larger effect sizes emerged, for which no physiological explanation could be derived from the current data.

## 7 Perspective

IHT has become very popular, and several studies have been published over the years. Although IHT was thought to promote greater adaptations than NT, our findings indicate that it does not enhance V̇O_2max_ and aerobic performance. Adding hypoxia during exercise should not be expected to produce superior adaptations to standard aerobic training. Therefore, given the practical challenges associated with hypoxic environments, IHT should be evaluated with the appropriate consideration.

## Author Contributions

G.D., M.V., F.S., and E.W. were responsible for idea conception. G.D., G.G., E.W., and M.V. collaborated on the literature review. G.D. and G.G. performed the meta‐analysis and produced the figures and tables. All authors collaborated on interpreting the results, and G.D., M.V., G.G., and E.W. collaborated in writing the major parts of the manuscript. All authors contributed to the article, approved the submitted version, and read and approved the final manuscript.

## Conflicts of Interest

The authors declare no conflicts of interest.

## Supporting information


Data S1:

Data S2:

Table S1:

Table S2:

Table S3:

## Data Availability

The data S1 that supports the findings of this study are available in the supplementary material of this article.

## References

[1] H. Roskamm , F. Landry , L. Samek , M. Schlager , H. Weidemann , and H. Reindell , “Effects of a Standardized Ergometer Training Program at Three Different Altitudes,” Journal of Applied Physiology 27 (1969): 840–847. 5353208 10.1152/jappl.1969.27.6.840

[2] B. D. Levine , “Intermittent Hypoxic Training: Fact and Fancy,” High Altitude Medicine & Biology 3 (2002): 177–193. 12162862 10.1089/15270290260131911

[3] H. Hoppeler , S. Klossner , and M. Vogt , “Training in Hypoxia and Its Effects on Skeletal Muscle Tissue,” Scandinavian Journal of Medicine & Science in Sports 18, no. Suppl 1 (2008): 38–49. 18665951 10.1111/j.1600-0838.2008.00831.x

[4] R. Faiss , O. Girard , and G. P. Millet , “Advancing Hypoxic Training in Team Sports: From Intermittent Hypoxic Training to Repeated Sprint Training in Hypoxia,” British Journal of Sports Medicine 47 (2013): i45–i50. 24282207 10.1136/bjsports-2013-092741 PMC3903143

[5] J. A. L. Calbet , G. Rådegran , R. Boushel , and B. Saltin , “On the Mechanisms That Limit Oxygen Uptake During Exercise in Acute and Chronic Hypoxia: Role of Muscle Mass,” Journal of Physiology 587 (2009): 477–490. 19047206 10.1113/jphysiol.2008.162271 PMC2670057

[6] J. E. Peltonen , H. O. Tikkanen , and H. K. Rusko , “Cardiorespiratory Responses to Exercise in Acute Hypoxia, Hyperoxia and Normoxia,” European Journal of Applied Physiology 85 (2001): 82–88. 11513325 10.1007/s004210100411

[7] P. D. Wagner , “A Theoretical Analysis of Factors Determining V̇O2max at Sea Level and Altitude,” Respiration Physiology 106 (1996): 329–343. 9017851 10.1016/s0034-5687(96)00086-2

[8] S. P. Dufour , E. Ponsot , J. Zoll , et al., “Exercise Training in Normobaric Hypoxia in Endurance Runners. I. Improvement in Aerobic Performance Capacity,” Journal of Applied Physiology (1985) 100 (2006): 1238–1248. 10.1152/japplphysiol.00742.2005 16540709

[9] M. Czuba , O. Fidos‐Czuba , K. Płoszczyca , A. Zajac , and J. Langfort , “Comparison of the Effect of Intermittent Hypoxic Training vs. the Live High, Train Low Strategy on Aerobic Capacity and Sports Performance in Cyclists in Normoxia,” Biology of Sport 35 (2018): 39–48. 30237660 10.5114/biolsport.2018.70750 PMC6135973

[10] M. Czuba , R. Wilk , J. Karpiński , M. Chalimoniuk , A. Zajac , and J. Langfort , “Intermittent Hypoxic Training Improves Anaerobic Performance in Competitive Swimmers When Implemented Into a Direct Competition Mesocycle,” PLoS One 12 (2017): e0180380. 28763443 10.1371/journal.pone.0180380 PMC5538675

[11] M. Czuba , Z. Waskiewicz , A. Zajac , S. Poprzecki , J. Cholewa , and R. Roczniok , “The Effects of Intermittent Hypoxic Training on Aerobic Capacity and Endurance Performance in Cyclists,” Journal of Sports Science & Medicine 10 (2011): 175–183. 24149312 PMC3737917

[12] M. Czuba , A. Zaja̧c , A. Maszczyk , et al., “The Effects of High Intensity Interval Training in Normobaric Hypoxia on Aerobic Capacity in Basketball Players,” Journal of Human Kinetics 39 (2013): 103–114. 24511346 10.2478/hukin-2013-0073 PMC3916912

[13] H. Y. Park and K. Lim , “Effects of Hypoxic Training Versus Normoxic Training on Exercise Performance in Competitive Swimmers,” Journal of Sports Science and Medicine 16 (2017): 480–488. 29238247 PMC5721177

[14] H. Y. Park , W. S. Jung , S. W. Kim , and K. Lim , “Effects of Interval Training Under Hypoxia on the Autonomic Nervous System and Arterial and Hemorheological Function in Healthy Women,” International Journal of Women's Health 14 (2022): 79–90. 10.2147/IJWH.S344233 PMC8818981 35140525

[15] W. S. Jung , S. W. Kim , and H. Y. Park , “Interval Hypoxic Training Enhances Athletic Performance and Does Not Adversely Affect Immune Function in Middle‐ and Long‐Distance Runners,” International Journal of Environmental Research and Public Health 17 (2020): 1934. 32188027 10.3390/ijerph17061934 PMC7143158

[16] G. Millet , D. J. Bentley , B. Roels , L. R. Mc Naughton , J. Mercier , and D. Cameron‐Smith , “Effects of Intermittent Training on Anaerobic Performance and MCT Transporters in Athletes,” PLoS One 9 (2014): e95092. 24797797 10.1371/journal.pone.0095092 PMC4010422

[17] B. Roels , D. J. Bentley , O. Coste , J. Mercier , and G. P. Millet , “Effects of Intermittent Hypoxic Training on Cycling Performance in Well‐Trained Athletes,” European Journal of Applied Physiology 101 (2007): 359–368. 17636319 10.1007/s00421-007-0506-8

[18] T. Debevec , M. Amon , M. E. Keramidas , S. N. Kounalakis , R. Pišot , and I. B. Mekjavic , “Normoxic and Hypoxic Performance Following 4 Weeks of Normobaric Hypoxic Training,” Aviation, Space, and Environmental Medicine 81 (2010): 387–393. 20377142 10.3357/asem.2660.2010

[19] B. A. Holliss , R. J. Burden , A. M. Jones , and C. R. Pedlar , “Eight Weeks of Intermittent Hypoxic Training Improves Submaximal Physiological Variables in Highly Trained Runners,” Journal of Strength and Conditioning Research 28 (2014): 2195–2203. 24513622 10.1519/JSC.0000000000000406

[20] D. L. Bonetti and W. G. Hopkins , “Sea‐Level Exercise Performance Following Adaptation to Hypoxia A Meta‐Analysis,” Sports Medicine 39 (2009): 107–127. 19203133 10.2165/00007256-200939020-00002

[21] D. Montero and C. Lundby , “Effects of Exercise Training in Hypoxia Versus Normoxia on Vascular Health,” Sports Medicine 46 (2016): 1725–1736. 27286988 10.1007/s40279-016-0570-5

[22] A. Westmacott , N. E. M. Sanal‐Hayes , M. McLaughlin , J. L. Mair , and L. D. Hayes , “High‐Intensity Interval Training (HIIT) in Hypoxia Improves Maximal Aerobic Capacity More Than HIIT in Normoxia: A Systematic Review, Meta‐Analysis, and Meta‐Regression,” International Journal of Environmental Research and Public Health 19 (2022): 14261. 36361141 10.3390/ijerph192114261 PMC9658399

[23] X. Feng , L. Zhao , Y. Chen , Z. Wang , H. Lu , and C. Wang , “Optimal Type and Dose of Hypoxic Training for Improving Maximal Aerobic Capacity in Athletes: A Systematic Review and Bayesian Model‐Based Network Meta‐Analysis,” Frontiers in Physiology 14 (2023): 1–10. 10.3389/fphys.2023.1223037 PMC10513096 37745240

[24] Q. Yu , Z. Kong , L. Zou , R. Chapman , Q. Shi , and J. Nie , “Comparative Efficacy of Various Hypoxic Training Paradigms on Maximal Oxygen Consumption: A Systematic Review and Network Meta‐Analysis,” Journal of Exercise Science and Fitness 21 (2023): 366–375. 37854170 10.1016/j.jesf.2023.09.001 PMC10580050

[25] J. P. T. Higgins , J. Thomas , J. Chandler , M. Cumpston , T. Li , and M. J. Page , eds., Cochrane Handbook for Systematic Reviews of Interventions Version 6.4 (Cochrane, 2023).

[26] O. Girard , F. Brocherie , and G. P. Millet , “Effects of Altitude/Hypoxia on Single‐ and Multiple‐Sprint Performance: A Comprehensive Review,” Sports Medicine 47 (2017): 1931–1949. 28451905 10.1007/s40279-017-0733-z

[27] F. Brocherie , O. Girard , R. Faiss , and G. P. Millet , “Effects of Repeated‐Sprint Training in Hypoxia on Sea‐Level Performance: A Meta‐Analysis,” Sports Medicine 47 (2017): 1651–1660. 28194720 10.1007/s40279-017-0685-3

[28] G. P. Millet , R. Faiss , F. Brocherie , and O. Girard , “Hypoxic Training and Team Sports: A Challenge to Traditional Methods?,” British Journal of Sports Medicine 47 (2013): i6–i7. 24282210 10.1136/bjsports-2013-092793 PMC3903151

[29] M. Buchheit and P. B. Laursen , “High‐Intensity Interval Training, Solutions to the Programming Puzzle,” Sports Medicine 43 (2013): 313–338. 23539308 10.1007/s40279-013-0029-x

[30] J. A. Hawley and T. D. Noakes , Peak Power Output Predicts Maximal Oxygen Uptake and Performance Time in Trained Cyclists (Springer‐Verlag, 1992), 79–83. 10.1007/BF01466278 1505544

[31] L. S. Pescatello , R. Arena , D. Riebe , and P. D. Thompson , ACSM'S Guidelines for Exercise Testing andPrescription. 9th Edition (Wolters Kluwer/Lippincott Williams & Wilkins Health, 2014).

[32] B. Van Hooren , T. Souren , and B. C. Bongers , “Accuracy of Respiratory Gas Variables, Substrate, and Energy Use From 15 CPET Systems During Simulated and Human Exercise,” Scandinavian Journal of Medicine & Science in Sports 34 (2024): e14490. 37697640 10.1111/sms.14490

[33] L. V. Hedges , “Distribution Theory for Glass's Estimator of Effect Size and Related Estimators,” Journal of Educational Statistics 6 (1981): 107–128.

[34] W. G. Hopkins , S. W. Marshall , A. M. Batterham , and J. Hanin , “Progressive Statistics for Studies in Sports Medicine and Exercise Science,” Medicine and Science in Sports and Exercise 41 (2009): 3–12. 19092709 10.1249/MSS.0b013e31818cb278

[35] J. P. T. Higgins , S. G. Thompson , J. J. Deeks , and D. G. Altman , “Measuring Inconsistency in Meta‐Analyses,” British Medical Journal 327 (2003): 557–560. 12958120 10.1136/bmj.327.7414.557 PMC192859

[36] S. Nakagawa , Y. Yang , E. L. Macartney , R. Spake , and M. Lagisz , “Quantitative Evidence Synthesis: A Practical Guide on Meta‐Analysis, Meta‐Regression, and Publication Bias Tests for Environmental Sciences,” Environmental Evidence 12 (2023): 1–19. 39294795 10.1186/s13750-023-00301-6 PMC11378872

[37] M. Egger , G. D. Smith , and A. N. Phillips , “Meta‐Analysis: Principles and Procedures,” BMJ 315 (1997): 1533–1537. 9432252 10.1136/bmj.315.7121.1533 PMC2127925

[38] W. Viechtbauer and M. W. L. Cheung , “Outlier and Influence Diagnostics for Meta‐Analysis,” Research Synthesis Methods 1 (2010): 112–125. 26061377 10.1002/jrsm.11

[39] F. Yates , “Contingency Tables Involving Small Numbers and the χ2 Test,” Journal of the Royal Statistical Society. Series B, Statistical Methodology 1 (1934): 217–235.

[40] D. Desplanches , H. Hoppeler , M. T. Linossier , et al., “Effects of Training in Normoxia and Normobaric Hypoxia on Human Muscle Ultrastructure,” Pflügers Archiv 425 (1993): 263–267. 8309787 10.1007/BF00374176

[41] K. Engfred , M. Kjær , N. H. Secher , et al., “Hypoxia and Training‐Induced Adaptation of Hormonal Responses to Exercise in Humans,” European Journal of Applied Physiology and Occupational Physiology 68 (1994): 303–309. 8055887 10.1007/BF00571448

[42] D. L. Emonson , A. H. K. Aminuddin , R. L. Wight , G. C. Scroop , and C. J. Gore , “Training‐Induced Increases in Sea Level V̇O2max and Endurance Are Not Enhanced by Acute Hypobaric Exposure,” European Journal of Applied Physiology and Occupational Physiology 76 (1997): 8–12. 9243164 10.1007/s004210050206

[43] K. Katayama , Y. Sato , Y. Morotome , et al., “Ventilatory Chemosensitive Adaptations to Intermittent Hypoxic Exposure With Endurance Training and Detraining,” Journal of Applied Physiology (Bethesda, Md.: 1985) 86 (1999): 1805–1811. 10368341 10.1152/jappl.1999.86.6.1805

[44] D. M. Bailey , B. Davies , and J. Baker , “Training in Hypoxia: Modulation of Metabolic and Cardiovascular Risk Factors in Men,” Medicine and Science in Sports and Exercise 32 (2000): 1058–1066. 10862530 10.1097/00005768-200006000-00004

[45] J. Geiser , M. Vogt , R. Billeter , C. Zuleger , F. Belforti , and H. Hoppeler , “Training High ‐ Living Low: Changes of Aerobic Performance and Muscle Structure With Training at Simulated Altitude,” International Journal of Sports Medicine 22 (2001): 579–585. 11719893 10.1055/s-2001-18521

[46] M. Vogt , A. Puntschart , J. Geiser , C. Zuleger , R. Billeter , and H. Hoppeler , “Molecular Adaptations in Human Skeletal Muscle to Endurance Training Under Simulated Hypoxic Conditions,” Journal of Applied Physiology 91 (2001): 173–182. 11408428 10.1152/jappl.2001.91.1.173

[47] K. Masuda , K. Okazaki , S. Kuno , K. Asano , H. Shimojo , and S. Katsuta , “Endurance Training Under 2500‐m Hypoxia Does Not Increase Myoglobin Content in Human Skeletal Muscle,” European Journal of Applied Physiology 85 (2001): 486–490. 11606019 10.1007/s004210100471

[48] T. Meeuwsen , I. J. M. Hendriksen , and M. Holewijn , “Training‐Induced Increases in Sea‐Level Performance Are Enhanced by Acute Intermittent Hypobaric Hypoxia,” European Journal of Applied Physiology 84 (2001): 283–290. 11374111 10.1007/s004210000363

[49] L. Messonnier , H. Freund , L. Féasson , et al., “Blood Lactate Exchange and Removal Abilities After Relative High‐Intensity Exercise: Effects of Training in Normoxia and Hypoxia,” European Journal of Applied Physiology 84 (2001): 403–412. 11417427 10.1007/s004210000378

[50] N. Ventura , H. Hoppeler , R. Seiler , et al., “The Response of Trained Athletes to Six Weeks of Endurance Training in Hypoxia or Normoxia,” International Journal of Sports Medicine 24 (2003): 166–172. 12740733 10.1055/s-2003-39086

[51] L. Messonnier , A. Geyssant , F. Hintzy , and J. R. Lacour , “Effects of Training in Normoxia and Normobaric Hypoxia on Time to Exhaustion at the Maximum Rate of Oxygen Uptake,” European Journal of Applied Physiology 92 (2004): 470–476. 15138836 10.1007/s00421-004-1117-2

[52] J. P. Morton and N. T. Cable , “The Effects of Intermittent Hypoxic Training on Aerobic and Anaerobic Performance,” Ergonomics 48 (2005): 1535–1546. 16338719 10.1080/00140130500100959

[53] E. Ponsot , S. P. Dufour , J. Zoll , et al., “Exercise Training in Normobaric Hypoxia in Endurance Runners. II. Improvement of Mitochondrial Properties in Skeletal Muscle,” Journal of Applied Physiology (1985) 100 (2006): 1249–1257. 10.1152/japplphysiol.00361.2005 16339351

[54] J. Zoll , E. Ponsot , S. Dufour , et al., “Exercise Training in Normobaric Hypoxia in Endurance Runners. III. Muscular Adjustments of Selected Gene Transcripts,” Journal of Applied Physiology (1985) 100 (2006): 1258–1266. 10.1152/japplphysiol.00359.2005 16540710

[55] J. S. S. Wang , M. H. H. Wu , T. Y. Y. Mao , T. c. C. Fu , and C. C. C. Hsu , “Effects of Normoxic and Hypoxic Exercise Regimens on Cardiac, Muscular, and Cerebral Hemodynamics Suppressed by Severe Hypoxia in Humans,” Journal of Applied Physiology (1985) 109 (2010): 219–229. 10.1152/japplphysiol.00138.2010 20431021

[56] J. S. Wang , W. L. Chen , and T. P. Weng , “Hypoxic Exercise Training Reduces Senescent T‐Lymphocyte Subsets in Blood,” Brain, Behavior, and Immunity 25 (2011): 270–278. 20884344 10.1016/j.bbi.2010.09.018

[57] J. S. Wang , Y. C. Chen , W. L. Chen , and C. P. Lin , “Effects of Normoxic and Hypoxic Exercise Regimens on Lymphocyte Apoptosis Induced by Oxidative Stress in Sedentary Males,” European Journal of Applied Physiology 117 (2017): 2445–2455. 28988307 10.1007/s00421-017-3731-9

[58] Y. C. Chen , W. Y. Chou , T. C. Fu , and J. S. Wang , “Effects of Normoxic and Hypoxic Exercise Training on the Bactericidal Capacity and Subsequent Apoptosis of Neutrophils in Sedentary Men,” European Journal of Applied Physiology 118 (2018): 1985–1995. 29987365 10.1007/s00421-018-3935-7

[59] D. Desplanches , M. Amami , S. Dupré‐Aucouturier , et al., “Hypoxia Refines Plasticity of Mitochondrial Respiration to Repeated Muscle Work,” European Journal of Applied Physiology 114 (2014): 405–417. 24327174 10.1007/s00421-013-2783-8 PMC3895187

[60] A. M. J. J. Sanchez and F. Borrani , “Effects of Intermittent Hypoxic Training Performed at High Hypoxia Level on Exercise Performance in Highly Trained Runners,” Journal of Sports Sciences 36 (2018): 2045–2052. 29394148 10.1080/02640414.2018.1434747

[61] S. W. Kim , W. S. Jung , J. W. Kim , S. S. Nam , and H. Y. Park , “Aerobic Continuous and Interval Training Under Hypoxia Enhances Endurance Exercise Performance With Hemodynamic and Autonomic Nervous System Function in Amateur Male Swimmers,” International Journal of Environmental Research and Public Health 18 (2021): 3944. 33918616 10.3390/ijerph18083944 PMC8068973

[62] C. L. Lin , J. S. Wang , T. C. Fu , C. C. Hsu , and Y. C. Huang , “Hypoxic Exercise Training Elevates Erythrocyte Aggregation,” Applied Sciences 11 (2021): 6038.

[63] H. Y. Park , W. S. Jung , S. W. Kim , J. Kim , and K. Lim , “Effects of Interval Training Under Hypoxia on Hematological Parameters, Hemodynamic Function, and Endurance Exercise Performance in Amateur Female Runners in Korea,” Frontiers in Physiology 13 (2022): 1–11. 10.3389/fphys.2022.919008 PMC9158122 35665230

[64] D. E. R. Warburton , M. J. F. Haykowsky , H. A. Quinney , D. P. Humen , and K. K. Teo , “Reliability and Validity of Measures of Cardiac Output During Incremental to Maximal Aerobic Exercise Part I: Conventional Techniques,” Sports Medicine 27 (1999): 23–41. 10028131 10.2165/00007256-199927010-00003

[65] P. Robach , T. Bonne , D. Flück , et al., “Hypoxic Training: Effect on Mitochondrial Function and Aerobic Performance in Hypoxia,” Medicine and Science in Sports and Exercise 46 (2014): 1936–1945. 24674976 10.1249/MSS.0000000000000321

[66] N. Terrados , E. Jansson , C. Sylven , and L. Kaijser , “Is Hypoxia a Stimulus for Synthesis of Oxidative Enzymes and Myoglobin?,” Journal of Applied Physiology 68 (1990): 2369–2372. 2384418 10.1152/jappl.1990.68.6.2369

[67] N. Terrados , J. Melichna , C. Sylvén , E. Jansson , and L. Kaijser , “Effects of Training at Simulated Altitude on Performance and Muscle Metabolic Capacity in Competitive Road Cyclists,” European Journal of Applied Physiology and Occupational Physiology 57 (1988): 203–209. 3349988 10.1007/BF00640664

[68] J. Myers , M. Prakash , V. Froelicher , D. Do , S. Partington , and J. E. Atwood , “Exercise Capacity and Mortality Among Men Referred for Exercise Testing,” New England Journal of Medicine 346 (2002): 793–801. 11893790 10.1056/NEJMoa011858

[69] N. S. Murali , H. R. Murali , P. Auethavekiat , et al., “Impact of FUTON and NAA Bias on Visibility of Research,” Mayo Clinic Proceedings 79 (2004): 1001–1006. 15301326 10.4065/79.8.1001

[70] R. Wentz , “Visibility of Research: FUTON Bias,” Lancet 360 (2002): 1256. 12401287 10.1016/S0140-6736(02)11264-5

