# Supplementary material for: Aerobic Intermittent Hypoxic Training Is Not Beneficial for Maximal Oxygen Uptake and Performance: A Systematic Review and Meta‐Analysis
Source: Scand J Med Sci Sports. 2025 Jun 23;35(6):e70088. doi: 10.1111/sms.70088 (PMC12184621; doi:10.1111/sms.70088)
Supplement: Supplementary file 2 — Data S2: [file SMS-35-e70088-s005.docx]

**Aerobic Intermittent Hypoxic Training is Not Beneficial for Maximal Oxygen Uptake and Performance: A Systematic Review and Meta-Analysis**

**1. Supplementary Information: Methods**

*1.1 Definition of the Studies with Physiologically Inconsistent Results*

Each study was carefully screened for methodological issues that could influence the overall effect size of the meta-analysis. Considering the well-established relationship between maximal workload and V̇O_2max_ ^1,2^ — and considering that standard aerobic training predominantly enhances oxygen transport — study results displaying substantial divergence between these two maximal values should raise suspicions of methodological issues ^3^. While improvements in maximal workload can occur without corresponding gains in V̇O_2max_, such cases are attributable to enhanced exercise efficiency or a greater reliance on anaerobic energy contribution to maintain a V̇O_2_ plateau ^3^.

To identify inconsistent results, we employed a three-phase process: 1) Pre-post changes of the normoxic training group (NT) were standardized using Cohen’s d ^4^; 2) Studies presenting physiologically implausible outcomes were identified. These included instances in which changes in V̇O_2max_ and peak power output (PPO) or maximal running speed were discordant (*e.g.*, a significant decrease in V̇O_2max_ alongside a significant increase in PPO, or *vice versa*), or where a substantial improvement in maximal workload occurred in the absence of a meaningful change in V̇O_2max_; 3) All flagged studies were independently re-evaluated in a blinded manner by three authors (GD, MV, EW) to assess whether the study design and results could plausibly explain the observed discrepancies. If no adequate explanation was identified, the study was excluded from further analysis.

*1.2 Definition of Discrete Moderators*

Moderators included characteristics of the population, training, hypoxia administration, maximal testing methodologies, experimental design, and publication features. The definitions of all the discrete moderators are provided in Supplementary Information Table 1.

*1.3 Definition of Continuous Moderators*

Continuous moderators for meta-regression analysis were: 1) Fraction of Inspired Oxygen (FiO_2_, %), 2) Simulated Altitude (meters above sea level, m asl); 3) weeks of training (w); 4) total day of session (days); 5) total time spent doing IHT/NT (min); 6) frequency of days/week; 7) average minutes/week; 8) average minutes/day; 9) year of publication.

*1.4 Calculation of Absolute V̇o_2max_*

The absolute values of maximal oxygen uptake (_abs_V̇O_2max_) were estimated using body weight (BW) variations, for studies that reported only relative V̇O_2max_ (ml⋅kg^-1^⋅min^-1^) mean (μ) and standard deviation (SD). If pre- and post-intervention BW μ and SD were available for both groups, to estimate _abs_V̇O_2max_ (ml⋅min^-1^), the formula for the product of independent variables was used ^5^:

1) μ _abs_V̇O_2max_ = μV̇O_2max_ ⋅ μBW;

2) SD _abs_V̇O_2max_ = √((μBW^2^⋅SDV̇O_2max_^2^) + (μV̇O_2max_^2^⋅SDBW^2^) + (SD BW^2^⋅SDV̇O_2max_^2^))

**2. Supplementary Information: Results**

*2.1 Papers Excluded for Physiologically Inconsistent Results*

Three papers showed physiologically inconsistent results that contradict established principles and were excluded from the analysis. In particular, NT groups in the papers by Ramos-Campo *et al.* ^6^ and Czuba *et al.* ^7^, showed a significant decrease in V̇O_2max_ in endurance athletes after training, despite a significant increase in maximal workload. Such discrepancies raise concerns regarding the methodological integrity of V̇O_2max_ assessments.

Similarly, the study by Haufe *et al.* 2008 ^8^, which investigated healthy sedentary individuals, reported no change in V̇O_2max_ in the NT group (*d* = 0.00), despite a marked increase in time to exhaustion during maximal testing (*d* = 1.02). While this suggested improved running efficiency, it remains unclear why the improvement in maximal test performance was comparable between the NT and IHT groups, considering that V̇O_2max_ increased only in IHT. Furthermore, if enhanced anaerobic capacity had contributed to a better ability to sustain a V̇O_2max_ plateau, one would expect an accompanying rise in maximal blood lactate concentrations. However, the study reported a decrease in peak lactate from 13 to 11 mmol∙L^-1^ following training. Collectively, these data raise concerns about the accuracy of V̇O_2max_ measurement in this study.

*2.2 Studies with Multiple Groups, Overlapping Cohorts, and Computation of the Reports*

The studies by Engfred *et al.* ^9^, Geiser *et al.* ^10^, Vogt *et al.* ^11^, and the three by Wang *et al.* ^12–14^ were considered multiple reports. Overlapping participant cohorts are declared in the studies from Dufour *et al.* ^15^, Ponsot *et al.* ^16^, and Zoll *et al.* ^17^. High probability of overlapping participant cohorts is present in the studies by Geiser *et al.* ^10^, Vogt *et al.* ^11^, in the three studies by Wang *et al.* ^12–14^ and Chen *et al.* ^18^, as well as in two studies by Park *et al.* ^19,20^. Therefore, we considered these 11 studies as 6 reports corresponding to 6 different cohorts.

Supplementary Information Figure 1 provides a detailed description of the cohort (report) computation for the analysis.

**3. References**

1. Noakes TD, Myburgh KH, Schall R. Peak treadmill running velocity during the vo2 max test predicts running performance. J Sports Sci 1990;8:35–45.

2. Hawley JA, Noakes TD. Peak power output predicts maximal oxygen uptake and performance time in trained cyclists. Springer-Verlag; 1992. 79–83 p.

3. Buchheit M, Laursen PB. High-intensity interval training, solutions to the programming puzzle: Part I: Cardiopulmonary emphasis. Sports Medicine 2013;43:313–338.

4. Fritz CO, Morris PE, Richler JJ. Effect size estimates: Current use, calculations, and interpretation. J Exp Psychol Gen 2012;141:2–18.

5. Barnett HAR. The Variance of the Product of Two Independent Variables and Its Application to an Investigation Based on Sample Data. J Inst Actuar 1955;81:190–190.

6. Ramos-Campo DJ, Martínez-Sánchez F, Esteban-García P, Rubio-Arias JA, Clemente-Suarez VJ, Jiménez-Díaz JF. The effects of intermittent hypoxia training on hematological and aerobic performance in triathletes. Acta Physiol Hung 2015;102:409–418.

7. Czuba M, Bril G, Płoszczyca K, Piotrowicz Z, Chalimoniuk M, Roczniok R, Zembroń-ŁAcny A, Gerasimuk D, Langfort J. Intermittent hypoxic training at lactate threshold intensity improves aiming performance in well-trained biathletes with little change of cardiovascular variables. Biomed Res Int 2019;2019:.

8. Haufe S, Wiesner S, Engeli S, Luft FC, Jordan J. Influences of Normobaric Hypoxia Training on Metabolic Risk Markers in Human Subjects. Med Sci Sports Exerc 2008;40:1939–1944.

9. Engfred K, Kjær M, Secher NH, Friedman DB, Hanel B, Nielsen OJ, Bach FW, Galbo H, Levine BD. Hypoxia and training-induced adaptation of hormonal responses to exercise in humans. Eur J Appl Physiol Occup Physiol 1994;68:303–309.

10. Geiser J, Vogt M, Billeter R, Zuleger C, Belforti F, Hoppeler H. Training high - Living low: Changes of aerobic performance and muscle structure with training at simulated altitude. Int J Sports Med 2001;22:579–585.

11. Vogt M, Puntschart A, Geiser J, Zuleger C, Billeter R, Hoppeler H. Molecular adaptations in human skeletal muscle to endurance training under simulated hypoxic conditions. J Appl Physiol 2001;91:173–182.

12. Wang JSS, Wu MHH, Mao TYY, Fu T cheng C, Hsu CCC. Effects of normoxic and hypoxic exercise regimens on cardiac, muscular, and cerebral hemodynamics suppressed by severe hypoxia in humans. J Appl Physiol 2010;109:219–229.

13. Wang JS, Chen WL, Weng TP. Hypoxic exercise training reduces senescent T-lymphocyte subsets in blood. Brain Behav Immun 2011;25:270–278.

14. Wang JS, Chen YC, Chen WL, Lin CP. Effects of normoxic and hypoxic exercise regimens on lymphocyte apoptosis induced by oxidative stress in sedentary males. Eur J Appl Physiol 2017;117:2445–2455.

15. Dufour SP, Ponsot E, Zoll J, Doutreleau S, Lonsdorfer-Wolf E, Geny B, Lampert E, Flü ck M, Hoppeler H, Billat V, Mettauer B, Richard R, Lonsdorfer J, Fluck M, Mettauer B trand. Exercise training in normobaric hypoxia in endurance runners. I. Improvement in aerobic performance capacity. J Appl Physiol 2006;100:1238–1248.

16. Ponsot E, Dufour SP, Zoll J, Doutrelau S, N’Guessan B, Geny B, Hoppeler H, Lampert E, Mettauer B, Ventura-Clapier R, Richard R. Exercise training in normobaric hypoxia in endurance runners. II. Improvement of mitochondrial properties in skeletal muscle. J Appl Physiol (1985) 2006;100:1249–1257.

17. Zoll J, Ponsot E, Dufour S, Doutreleau S, Ventura-Clapier R, Vogt M, Hoppeler H, Richard R, Flück M. Exercise training in normobaric hypoxia in endurance runners. III. Muscular adjustments of selected gene transcripts. J Appl Physiol (1985) 2006;100:1258–1266.

18. Chen YC, Chou WY, Fu TC, Wang JS. Effects of normoxic and hypoxic exercise training on the bactericidal capacity and subsequent apoptosis of neutrophils in sedentary men. Eur J Appl Physiol 2018;118:1985–1995.

19. Park HY, Jung WS, Kim SW, Kim J, Lim K. Effects of Interval Training Under Hypoxia on Hematological Parameters, Hemodynamic Function, and Endurance Exercise Performance in Amateur Female Runners in Korea. Front Physiol 2022;13:1–11.

20. Park HY, Jung WS, Kim SW, Lim K. Effects of Interval Training Under Hypoxia on the Autonomic Nervous System and Arterial and Hemorheological Function in Healthy Women. Int J Womens Health 2022;14:79–90.

**Supplementary Information Table 1.** Detailed description of the discrete moderators.

|  | **Levels for Moderator Analysis and Definitions** | |
| --- | --- | --- |
| **Population Characteristics** | |  |
| *Sex* | Female |  |
|  | Male |  |
|  | Mixed |  |
| *Athletic Background* | Cyclists |  |
|  | Other Endurance Athletes |  |
|  | Runners | |
|  | Sedentary Subjects |  |
|  | Swimmers |  |
|  | Team Sports Players |  |
|  | Triathletes |  |
| *Training Status* | Untrained |  |
|  | Competitive Athletes |  |
|  | Elite Athletes |  |
| *Country* | The country of the laboratory of the Corresponding Author was considered. Then, if a country has less than 3 reports for the analysis, it was pooled with other similar countries following a geographical distribution - *e.g.*, “Europe (Others)”, “Asia (Others)” | |
| **Training and Hypoxia Characteristics** | |  |
| *Training Schedule* | Exclusive: No other programmed sessions other than IHT/NT | |
|  | Integrated: IHT/NT was part of a broader training program | |
| *Exercise Intensity* | High Intensity (HIT): at least one IHT session per week at an intensity determined through one of the following methods: 1) the second ventilatory threshold (VT_2_); 2) the second lactate threshold (LT_2_, achieving a blood lactate level of 4 mmol∙L⁻¹); 3) 85% of the hypoxic/normoxic maximal heart rate (HR_max_)_,_ V̇O_2max_, or maximal workload. | |
|  | Moderate Intensity (MIT): other training not defined as HIT. | |
| *Type of Exercise* | Cycling | |
|  | Running |  |
|  | Mixed |  |
| *Method of Hypoxia* | Hypobaric Hypoxic Chamber (HHC) | |
|  | Normobaric Hypoxic Chamber (NHC) | |
|  | Hypoxicator (HD) | |
| *Intensity Matching between IHT and NT* | Same Relative Maximal Workload: the intensity was targeted through a percentage of hypoxic/normoxic maximal workload (PPO, V_peak_) | |
|  | Same Relative HR_max_/V̇O_2max_: the intensity was targeted through a percentage of hypoxic/normoxic HR_max,_ or V̇O_2max_ | |
|  | Absolute Normoxic Maximal Workload: the intensity was targeted only through a normoxic maximal workload (PPO, V_peak_) | |
|  | Absolute Normoxic HR_max_/V̇O_2max_: the intensity was targeted only through a percentage of normoxic HR_max,_ or V̇O_2max_ | |
| **V̇O_2max_ testing, criteria for maximal effort** |  |  |
| *Gas Sampling* | Breath-by-Breath |  |
|  | Douglas Bag |  |
|  | Mixing Chamber |  |
| *V̇O_2_ plateau criteria* | Yes |  |
|  | Not Specified |  |
| *RER value criteria* | Maximal effort considered with RER > 1.0 | |
|  | Maximal effort considered with RER > 1.1 | |
|  | Not Specified | |
| *La_max_ ≥ 8 mmol∙L^-1^ criteria* | Yes |  |
|  | Not Specified |  |
| *Encouragement* | Yes |  |
|  | Not Specified |  |
| **Publication Characteristics** | |  |
| *Randomization* | Randomized Clinical Trial, RCT |  |
|  | Not Specified - Clinical Trial, CT | |
| *Blind* | None (None, Not Specified) |  |
|  | Blind (Single, Double) |  |
| *Decade of Publication* | Before 2000 |  |
|  | 2000 - 2010 |  |
|  | After 2010 |  |
| *Open Access Publication* | Yes |  |
|  | No |  |

**Supplementary Information Figure 1**. A diagram showing the relationship between studies and reports considered for the meta-analysis
