# Supplementary material for: Aerobic Intermittent Hypoxic Training Is Not Beneficial for Maximal Oxygen Uptake and Performance: A Systematic Review and Meta‐Analysis
Source: Scand J Med Sci Sports. 2025 Jun 23;35(6):e70088. doi: 10.1111/sms.70088 (PMC12184621; doi:10.1111/sms.70088)
Supplement: Supplementary file 3 — Table S1: [file SMS-35-e70088-s004.docx]

**Supplementary Table 1.** Results from Leave-One-Out analysis for all the variables. Highly influential studies are marked in bold

| **Removed Study** | **SMD [95% CI]; *p*-value** | **Outlier** |
| --- | --- | --- |
| **Maximal Oxygen Uptake (V̇O_2max_)** |  |  |
| Desplanches D *et al.*, 1993 | 0.1 [-0.12, 0.33]; 0.352 | No |
| Engfred K *et al.* (Rel), 1994 | 0.11 [-0.12, 0.34]; 0.331 | No |
| Engfred K *et al.* (Abs), 1994 | 0.14 [-0.07, 0.35]; 0.171 | No |
| Emonson *et al.*, 1997 | 0.1 [-0.13, 0.33]; 0.363 | No |
| Katayama *et al.*, 1999 | 0.11 [-0.12, 0.33]; 0.347 | No |
| Bailey *et al.*, 2000 | 0.09 [-0.14, 0.32]; 0.434 | No |
| Geiser J *et al.* & Vogt M *et al.* (H), 2001 | 0.1 [-0.13, 0.33]; 0.378 | No |
| Geiser J *et al.* & Vogt M *et al.* (M), 2001 | 0.11 [-0.12, 0.34]; 0.336 | No |
| Masuda K *et al.*, 2001 | 0.12 [-0.11, 0.34]; 0.298 | No |
| Meeuwsen T *et al.*, 2001 | 0.11 [-0.12, 0.34]; 0.338 | No |
| Ventura N *et al.*, 2003 | 0.11 [-0.12, 0.34]; 0.337 | No |
| Messonnier L *et al.*, 2004 | 0.11 [-0.12, 0.33]; 0.347 | No |
| Morton JP & Cable NT, 2005 | 0.12 [-0.11, 0.34]; 0.306 | No |
| Dufour SP *et al.* & Zoll J *et al.* & Ponsot E *et al.*, 2006 | 0.10 [-0.13, 0.32]; 0.388 | No |
| Roels B *et al.*, 2007 | 0.17 [-0.04, 0.37]; 0.101 | No |
| Wang JS *et al.* (A), 2010 | 0.1 [-0.13, 0.32]; 0.389 | No |
| Wang JS *et al.* (B), 2010 | 0.13 [-0.1, 0.35]; 0.261 | No |
| Debevec T *et al.*, 2010 | 0.16 [-0.05, 0.37]; 0.133 | No |
| Czuba M *et al.*, 2011 | 0.09 [-0.14, 0.31]; 0.424 | No |
| Czuba M *et al.*, 2013 | 0.1 [-0.13, 0.33]; 0.375 | No |
| Holliss BA *et al.*, 2014 | 0.15 [-0.06, 0.36]; 0.16 | No |
| Millet G *et al.*, 2014 | 0.17 [-0.04, 0.37]; 0.101 | No |
| Desplanches D *et al.*, 2014 | 0.11 [-0.12, 0.33]; 0.343 | No |
| Czuba M *et al.*, 2017 | 0.1 [-0.12, 0.33]; 0.354 | No |
| Czuba M *et al.*, 2018 | 0.09 [-0.13, 0.32]; 0.414 | No |
| Jung W *et al.*, 2020 | 0.09 [-0.13, 0.32]; 0.414 | No |
| Kim *et al.*, 2021 | 0.09 [-0.13, 0.31]; 0.42 | No |
| Lin *et al.*, 2021 | 0.08 [-0.14, 0.31]; 0.452 | No |
| Park HY *et al.*, 2022 | 0.09 [-0.13, 0.32]; 0.416 | No |
|  |  |  |
| **Absolute Maximal Oxygen Uptake (_abs_V̇O_2max_)** |  |  |
| Desplanches D *et al.*, 1993 | 0.13 [-0.14, 0.4]; 0.322 | No |
| Engfred K *et al.* (Rel), 1994 | 0.14 [-0.13, 0.41]; 0.299 | No |
| Engfred K *et al.* (Abs), 1994 | 0.19 [-0.05, 0.42]; 0.117 | No |
| Emonson *et al.*, 1997 | 0.13 [-0.14, 0.4]; 0.328 | No |
| Bailey *et al.*, 2000 | 0.11 [-0.16, 0.38]; 0.411 | No |
| Masuda K *et al.*, 2001 | 0.15 [-0.12, 0.42]; 0.263 | No |
| Meeuwsen T *et al.*, 2001 | 0.14 [-0.13, 0.41]; 0.306 | No |
| Ventura N *et al.*, 2003 | 0.14 [-0.13, 0.41]; 0.304 | No |
| **Roels B *et al*.*, 2007*** | 0.22 [0.00, 0.44]; 0.048* | No |
| Wang JS *et al.* (A), 2010 | 0.12 [-0.15, 0.39]; 0.349 | No |
| Wang JS *et al.* (B), 2010 | 0.16 [-0.11, 0.43]; 0.226 | No |
| Czuba M *et al.*, 2011 | 0.11 [-0.15, 0.37]; 0.396 | No |
| Czuba M *et al.*, 2013 | 0.12 [-0.14, 0.39]; 0.346 | No |
| **Millet G *et al., 2014*** | 0.22 [0.00, 0.44]; 0.048* | No |
| Desplanches D *et al.*, 2014 | 0.14 [-0.13, 0.41]; 0.301 | No |
| Czuba M *et al.*, 2017 | 0.13 [-0.14, 0.4]; 0.324 | No |
| Czuba M *et al.*, 2018 | 0.11 [-0.15, 0.38]; 0.387 | No |
| Jung W *et al.*, 2020 | 0.12 [-0.15, 0.4]; 0.348 | No |
| Kim *et al.*, 2021 | 0.12 [-0.15, 0.39]; 0.355 | No |
| Lin *et al.*, 2021 | 0.1 [-0.17, 0.37]; 0.449 | No |
| Park HY *et al.*, 2022 | 0.12 [-0.15, 0.39]; 0.374 | No |
|  |  |  |
| **Peak Power Output (PPO)** |  |  |
| Bailey *et al.*, 2000 | 0.18 [-0.04, 0.4]; 0.101 | No |
| Geiser J *et al.* & Vogt M *et al.* (H), 2001 | 0.13 [-0.09, 0.35]; 0.221 | No |
| Geiser J *et al.* & Vogt M *et al.* (M), 2001 | 0.18 [-0.03, 0.38]; 0.094 | No |
| Meeuwsen T *et al.*, 2001 | 0.15 [-0.07, 0.37]; 0.166 | No |
| Ventura N *et al.*, 2003 | 0.14 [-0.08, 0.36]; 0.206 | No |
| Morton JP & Cable NT, 2005 | 0.16 [-0.06, 0.38]; 0.147 | No |
| Roels B *et al.*, 2007 | 0.17 [-0.05, 0.38]; 0.128 | No |
| Wang JS *et al.* (A), 2010 | 0.15 [-0.07, 0.37]; 0.17 | No |
| Wang JS *et al.* (B), 2010 | 0.13 [-0.09, 0.35]; 0.221 | No |
| Debevec T *et al.*, 2010 | 0.18 [-0.02, 0.39]; 0.08 | No |
| Czuba M *et al.*, 2011 | 0.1 [-0.1, 0.29]; 0.304 | No |
| Millet G *et al.*, 2014 | 0.17 [-0.05, 0.38]; 0.128 | No |
| Desplanches D *et al.*, 2014 | 0.16 [-0.06, 0.38]; 0.144 | No |
| Czuba M *et al.*, 2017 | 0.13 [-0.09, 0.34]; 0.233 | No |
| Czuba M *et al.*, 2018 | 0.11 [-0.1, 0.32]; 0.286 | No |
| Lin *et al.*, 2021 | 0.1 [-0.12, 0.32]; 0.353 | No |
|  |  |  |
| **Time-to-Exhaustion (TTE)** |  |  |
| Engfred K *et al.* (Rel), 1994 | -0.16 [-1.32, 1.01]; 0.695 | No |
| Engfred K *et al.* (Abs), 1994 | -0.2 [-1.43, 1.02]; 0.631 | No |
| Emonson *et al.*, 1997 | -0.48 [-1.75, 0.78]; 0.312 | No |
| Messonnier L *et al.*, 2004 | -0.16 [-1.38, 1.06]; 0.699 | No |
| Sanchez AMJ and Borrani F, 2018 | -0.53 [-1.51, 0.44]; 0.18 | No |
|  |  |  |
| **Capillary Length Density (CLD)** |  |  |
| Desplanches D *et al.*, 1993 | 0.33 [-0.77, 1.44]; 0.411 | No |
| Geiser J *et al.* & Vogt M *et al.* (H), 2001 | -0.08 [-1.18, 1.02]; 0.841 | No |
| Geiser J *et al.* & Vogt M *et al.* (M), 2001 | -0.03 [-1.24, 1.18]; 0.94 | No |
| Messonier *et al.*, 2001 | 0.19 [-1.19, 1.56]; 0.698 | No |
| Desplanches D *et al.*, 2014 | 0.31 [-0.91, 1.52]; 0.476 | No |
|  |  |  |
| **Subsarcolemmal Mitochondria (SSM)** |  |  |
| ***Desplanches D et al., 1993*** | 0.81 [0.11, 1.49]; 0.038* | No |
| Geiser J *et al.* & Vogt M *et al.* (H), 2001 | 0.24 [-2.1, 2.57]; 0.707 | No |
| Geiser J *et al.* & Vogt M *et al.* (M), 2001 | 0.4 [-2.22, 3.02]; 0.577 | No |
| Desplanches D *et al.*, 2014 | 0.27 [-2.06, 2.6]; 0.666 | No |
|  |  |  |
| **Citrate Synthase (CS)** |  |  |
| Masuda K *et al.*, 2001 | -0.41 [-2.7, 1.89]; 0.526 | No |
| Messonier *et al.*, 2001 | -0.18 [-2.62, 2.27]; 0.785 | No |
| Zoll J *et al.*, 2006 | -0.5 [-2.45, 1.45]; 0.385 | No |
| Roels B *et al.*, 2007 | 0.14 [-1.07, 1.34]; 0.67 | No |
|  |  |  |
| **Hemoglobin (Hb)** |  |  |
| Geiser J *et al.* & Vogt M *et al.* (H), 2001 | 0.11 [-0.06, 0.29]; 0.185 | No |
| Meeuwsen T *et al.*, 2001 | 0.12 [-0.06, 0.29]; 0.168 | No |
| Ventura N *et al.*, 2003 | 0.11 [-0.06, 0.29]; 0.176 | No |
| Morton JP & Cable NT, 2005 | 0.15 [-0.02, 0.32]; 0.087 | No |
| Dufour SP *et al.* & Zoll J *et al.* & Ponsot E *et al.*, 2006 | 0.13 [-0.05, 0.31]; 0.14 | No |
| Wang JS *et al.* (A), 2010 | 0.12 [-0.06, 0.29]; 0.174 | No |
| Wang JS *et al.* (B), 2010 | 0.11 [-0.06, 0.29]; 0.188 | No |
| Debevec T *et al.*, 2010 | 0.08 [-0.06, 0.23]; 0.238 | No |
| Czuba M *et al.*, 2011 | 0.13 [-0.05, 0.31]; 0.138 | No |
| Czuba M *et al.*, 2013 | 0.13 [-0.04, 0.31]; 0.13 | No |
| Czuba M *et al.*, 2017 | 0.11 [-0.06, 0.28]; 0.19 | No |
| ***Czuba M et al., 2018*** | 0.17 [0.01, 0.32]; 0.034* | No |
| Sanchez AMJ and Borrani F, 2018 | 0.12 [-0.06, 0.29]; 0.167 | No |
| Kim *et al.*, 2021 | 0.16 [0, 0.32]; 0.05 | No |
| Lin *et al.*, 2021 | 0.14 [-0.04, 0.32]; 0.122 | No |
| Park HY *et al.*, 2022 | 0.12 [-0.06, 0.3]; 0.176 | No |
|  |  |  |
| **Hematocrit (Ht)** |  |  |
| Meeuwsen T *et al.*, 2001 | 0.08 [-0.16, 0.33]; 0.467 | No |
| Ventura N *et al.*, 2003 | 0.07 [-0.17, 0.31]; 0.532 | No |
| Morton JP & Cable NT, 2005 | 0.09 [-0.15, 0.33]; 0.443 | No |
| Dufour SP *et al.* & Zoll J *et al.* & Ponsot E *et al.*, 2006 | 0.08 [-0.17, 0.32]; 0.503 | No |
| Wang JS *et al.* (A), 2010 | 0.05 [-0.18, 0.29]; 0.639 | No |
| Wang JS *et al.* (B), 2010 | 0.05 [-0.18, 0.29]; 0.637 | No |
| Debevec T *et al.*, 2010 | 0.06 [-0.18, 0.31]; 0.584 | No |
| Czuba M *et al.*, 2011 | 0.06 [-0.18, 0.3]; 0.601 | No |
| Czuba M *et al.*, 2013 | 0.09 [-0.16, 0.33]; 0.455 | No |
| Czuba M *et al.*, 2017 | 0.08 [-0.17, 0.32]; 0.515 | No |
| Czuba M *et al.*, 2018 | 0.1 [-0.15, 0.34]; 0.408 | No |
| Sanchez AMJ and Borrani F, 2018 | 0.07 [-0.18, 0.31]; 0.566 | No |
| Kim *et al.*, 2021 | 0.13 [-0.09, 0.35]; 0.226 | No |
| Lin *et al.*, 2021 | 0.18 [-0.03, 0.38]; 0.084 | No |
| Park HY *et al.*, 2022 | 0.03 [-0.19, 0.24]; 0.798 | No |
|  |  |  |

SMD: Standardized Mean Difference; CI: Confidence Interval
