# Supplementary material for: Aerobic Intermittent Hypoxic Training Is Not Beneficial for Maximal Oxygen Uptake and Performance: A Systematic Review and Meta‐Analysis
Source: Scand J Med Sci Sports. 2025 Jun 23;35(6):e70088. doi: 10.1111/sms.70088 (PMC12184621; doi:10.1111/sms.70088)
Supplement: Supplementary file 4 — Table S2: [file SMS-35-e70088-s002.docx]

**Supplementary Table 2.a.** Discrete Moderator Analysis for Maximal Oxygen Uptake (V̇O_2max_)

|  | ***k*** | **SMD [95% CI]; *p-value*** | **I^2^ (Q, *p-value*)** | **Trim and Fill**  **SMD [95% CI]** |
| --- | --- | --- | --- | --- |
| **Population Characteristics** |  |  |  |  |
| Sex (Q = 1.26, *p =* 0.530) |  |  |  |  |
| Male | 23 | 0.1 [-0.15, 0.36]; 0.401 | 28% (30.4, 0.109) | 0.1 [-0.15, 0.36] |
| Athletic Background  (Q = 8.98, *p =* 0.170) |  |  |  |  |
| Cyclists | 4 | 0.07 [-1.28, 1.42]; 0.878 | 65% (8.46, 0.038) | NA |
| Runners | 4 | 0.2 [-1.04, 1.45]; 0.639 | 50% (6.06, 0.109) | NA |
| Sedentary | 14 | 0.15 [-0.13, 0.43]; 0.272 | 0% (12.62, 0.478) | 0.15 [-0.13, 0.43] |
| Training Status (Q = 0.08, *p =* 0.780) |  |  |  |  |
| Untrained | 14 | 0.15 [-0.13, 0.43]; 0.272 | 0% (12.62, 0.478) | 0.15 [-0.13, 0.43] |
| Competitive | 15 | 0.09 [-0.28, 0.46]; 0.610 | 39% (23.02, 0.060) | 0.09 [-0.28, 0.46] |
| Country (Q = 27.35, *p <* 0.001*) |  |  |  |  |
| Asia (Other) | 6 | 0.22 [-0.12, 0.56]; 0.151 | 0% (2.54, 0.771) | NA |
| Europe (Other) | 7 | -0.31 [-1, 0.38]; 0.313 | 48% (11.49, 0.074) | NA |
| France | 4 | -0.08 [-1.29, 1.13]; 0.85 | 50% (6.03, 0.110) | NA |
| South Korea | 3 | 0.59 [0.53, 0.66]; 0.001* | 0% (0.01, 0.997) | NA |
| Poland | 4 | 0.49 [0.20, 0.80]; 0.013* | 0% (0.43, 0.934) | NA |
| Switzerland | 5 | -0.09 [-0.86, 0.69]; 0.776 | 29% (5.62, 0.229) | NA |
|  |  |  |  |  |
| **Training, Hypoxia Characteristics** |  |  |  |  |
| Training Schedule  (Q = 0.09, *p =* 0.760) |  |  |  |  |
| Exclusive | 20 | 0.14 [-0.12, 0.39]; 0.272 | 13% (21.92, 0.288) | 0.14 [-0.12, 0.39] |
| Integrated | 9 | 0.06 [-0.47, 0.59]; 0.806 | 41% (13.66, 0.091) | NA |
| Exercise Intensity  (Q = 0.19, *p =* 0.660) |  |  |  |  |
| High Intensity | 15 | 0.16 [-0.21, 0.52]; 0.374 | 41% (23.7, 0.052) | 0.16 [-0.21, 0.52] |
| Moderate Intensity | 14 | 0.06 [-0.22, 0.34]; 0.638 | 0% (11.48, 0.570) | 0.06 [-0.22, 0.34] |
| Type of Exercise  (Q = 1.77*,* *p =* 0.410) |  |  |  |  |
| Cycling | 23 | 0.05 [-0.19, 0.29]; 0.677 | 20% (27.46, 0.194) | 0.05 [-0.19, 0.29] |
| Running | 5 | 0.27 [-0.57, 1.11]; 0.426 | 35% (6.15, 0.189) | NA |
| Method of Hypoxia  (Q = 0.99*,* *p =* 0.610) |  |  |  |  |
| Hypobaric Hypoxic Chamber | 9 | 0.24 [-0.14, 0.62]; 0.179 | 0% (7.17, 0.518) | NA |
| Normobaric Hypoxic Chamber | 8 | 0.15 [-0.29, 0.58]; 0.452 | 18% (8.54, 0.288) | NA |
| Hypoxicator | 12 | -0.01 [-0.45, 0.42]; 0.946 | 42% (19.04, 0.060) | -0.01 [-0.45, 0.42] |
| Intensity Matching IHT *vs.* NT  (Q = 3.28*,* *p =* 0.350) |  |  |  |  |
| % Same Relative Workload | 10 | -0.18 [-0.74, 0.39]; 0.496 | 59% (22.16, 0.008) | -0.08 [-0.65, 0.49] |
| % of Normoxic Workload | 4 | 0.06 [-1.32; 1.44]; 0.774 | 51% (6.12, 0.110) | NA |
| % Same Relative HR_max_, or V̇O_2max_ | 11 | 0.28 [0.13, 0.42]; 0.002* | 0% (1.84, 0.997) | 0.35 [0.2, 0.5] |
| % of Normoxic HR_max_, or V̇O_2max_ | 4 | 0.27 [-0.42, 0.96]; 0.295 | 0% (2.39, 0.496) | NA |
|  |  |  |  |  |
| **V̇O_2max_** **Testing** |  |  |  |  |
| Gas Sampling (Q = 2.91*,* *p =* 0.230) |  |  |  |  |
| Breath-by-Breath | 21 | 0.16 [-0.1, 0.42]; 0.205 | 21% (25.34, 0.189) | 0.16 [-0.1, 0.42] |
| Douglas Bag | 6 | -0.17 [-0.92, 0.58]; 0.581 | 44% (8.97, 0.110) | NA |
| V̇O_2_ plateau criteria  (Q = 0.00*,* p > 0.999) |  |  |  |  |
| Yes | 14 | 0.11 [-0.22, 0.44]; 0.492 | 20% (16.27, 0.235) | 0.11 [-0.22, 0.44] |
| Not Specified | 15 | 0.11 [-0.23, 0.44]; 0.512 | 28% (19.36, 0.152) | 0.11 [-0.23, 0.44] |
| RER value criteria  (Q = 3.27*,* *p =* 0.190) |  |  |  |  |
| 1.0 | 8 | 0.18 [-0.07, 0.43]; 0.137 | 0% (2.23, 0.946) | NA |
| 1.1 | 6 | -0.36 [-1.21, 0.5]; 0.334 | 63% (13.47, 0.019*) | NA |
| Not Specified | 15 | 0.28 [0.02, 0.55]; 0.037* | 0% (13.58, 0.481) | 0.28 [0.02, 0.55] |
| La_max_ ≥ 8 mmol∙L^-1^ criteria  (Q = 0.79*,* *p =* 0.370) |  |  |  |  |
| Yes | 16 | 0.19 [-0.09, 0.49]; 0.165 | 8% (16.25, 0.366) | 0.2 [-0.09, 0.49] |
| Not Specified | 13 | 0.02 [-0.37, 0.38]; 0.979 | 36% (18.82, 0.093) | 0 [-0.37, 0.38] |
| Encouragement (Q = 0.07*,* *p =* 0.790) |  |  |  |  |
| Yes | 7 | 0.06 [-0.38, 0.5]; 0.744 | 0% (4.78, 0.573) | NA |
| Not Specified | 22 | 0.12 [-0.15, 0.39]; 0.371 | 32% (30.73, 0.078) | 0.12 [-0.15, 0.39] |
|  |  |  |  |  |
| **Publication Characteristics** |  |  |  |  |
| Randomization (Q = 0.01*,* *p =* 0.940) |  |  |  |  |
| None, or Not Specified | 7 | 0.09 [-0.41, 0.6]; 0.666 | 0% (5.55, 0.476) | NA |
| Yes | 22 | 0.11 [-0.15, 0.38]; 0.379 | 30% (30.08, 0.090) | 0.11 [-0.15, 0.38] |
| Blind (Q = 0.02*,* *p =* 0.902) |  |  |  |  |
| None, or Not Specified | 25 | 0.11 [-0.13, 0.35]; 0.341 | 21% (30.5, 0.169) | 0.11 [-0.13, 0.35] |
| Single, or Double-Blind | 4 | 0.07 [-1.02, 1.15]; 0.860 | 42% (5.17, 0.160) | NA |
| Decade of Publication  (Q = 1.18*,* *p =* 0.550) |  |  |  |  |
| Before 2000 | 5 | 0.02 [-0.73, 0.78]; 0.941 | 4% (4.17, 0.383) | NA |
| 2000 - 2010 | 13 | 0.0 [-0.31, 0.32]; 0.980 | 10% (13.37, 0.343) | 0 [-0.31, 0.32] |
| After 2010 | 11 | 0.26 [-0.17, 0.68]; 0.208 | 37% (15.86, 0.104) | 0.26 [-0.17, 0.68] |
| Open Access Publication  (Q = 3.43*,* *p =* 0.060) |  |  |  |  |
| No | 20 | -0.03 [-0.29, 0.23]; 0.831 | 10% (21, 0.337) | -0.03 [-0.29, 0.23] |
| Yes | 9 | 0.37 [-0.03, 0.78]; 0.068 | 22% (10.23, 0.249) | NA |
|  |  |  |  |  |

**Supplementary Table 2.b.** Discrete Moderator Analysis for Absolute V̇O_2max_ (_abs_V̇O_2max_)

|  | ***k*** | **SMD [95% CI]; *p-value*** | **I^2^ (Q, *p-value*)** | **Trim and Fill SMD [95% CI]** |
| --- | --- | --- | --- | --- |
| **Population Characteristics** |  |  |  |  |
| Sex (Q = 1.02*,* *p =* 0.60) |  |  |  |  |
| Male | 16 | 0.15 [-0.15, 0.45]; 0.294 | 25% (20.08, 0.169) | 0.15 [-0.15, 0.45] |
| Athletic Background  (Q = 10.58*,* *p =* 0.100) |  |  |  |  |
| Cyclists | 4 | 0.05 [-1.34, 1.45]; 0.912 | 66% (8.95, 0.030*) | NA |
| Sedentary | 9 | 0.23 [-0.12, 0.59]; 0.166 | 0% (7.44, 0.490) | NA |
| Training Status (Q = 0.39*,* *p =* 0.530) |  |  |  |  |
| Untrained | 9 | 0.23 [-0.12, 0.59]; 0.166 | 0% (7.44, 0.490) | NA |
| Competitive | 12 | 0.09 [-0.32, 0.49]; 0.648 | 36% (17.15, 0.104) | 0.09 [-0.32, 0.49] |
| Country (Q = 7.42*,* *p =* 0.190) |  |  |  |  |
| Asia (Other) | 5 | 0.24 [-0.2, 0.68]; 0.204 | 0% (2.57, 0.631) | NA |
| Europe (Other) | 4 | 0 [-1.22, 1.23]; 0.993 | 42% (5.15, 0.161) | NA |
| South Korea | 3 | 0.38 [0.17, 0.59]; 0.016* | 0% (0.07, 0.966) | NA |
| Poland | 4 | 0.5 [0.2, 0.80]; 0.013* | 0% (0.43, 0.934) | NA |
| Switzerland | 3 | -0.37 [-2.32, 1.58]; 0.497 | 50% (4.01, 0.135) | NA |
|  |  |  |  |  |
| **Training, Hypoxia Characteristics** |  |  |  |  |
| Training Schedule  (Q = 0.02*,* *p =* 0.890) |  |  |  |  |
| Exclusive | 15 | 0.16 [-0.14, 0.45]; 0.281 | 14% (16.28, 0.297) | 0.16 [-0.14, 0.45] |
| Integrated | 6 | 0.11 [-0.6, 0.82]; 0.699 | 42% (8.63, 0.125) | NA |
| Exercise Intensity  (Q = 0.00*,* *p =* 0.980) |  |  |  |  |
| High Intensity | 11 | 0.15 [-0.3, 0.59]; 0.479 | 44% (17.78, 0.059) | 0.15 [-0.3, 0.59] |
| Moderate Intensity | 10 | 0.14 [-0.18, 0.47]; 0.35 | 0% (7.14, 0.622) | 0.36 [0.01, 0.71] |
| Type of Exercise  (Q = 4.82*,* *p =* 0.090) |  |  |  |  |
| Cycling | 17 | 0.06 [-0.26, 0.38]; 0.713 | 32% (23.7, 0.096) | 0.06 [-0.26, 0.38] |
| Running | 3 | 0.41 [0.17, 0.65]; 0.018* | 0% (0.08, 0.962) | NA |
| Method of Hypoxia  (Q = 2.55*,* *p =* 0.280) |  |  |  |  |
| Hypobaric Hypoxic Chamber | 8 | 0.15 [-0.23, 0.52]; 0.394 | 0% (5.43, 0.608) | NA |
| Normobaric Hypoxic Chamber | 6 | 0.36 [0.04, 0.69]; 0.035* | 0% (2.32, 0.803) | NA |
| Hypoxicator | 7 | -0.1 [-0.83, 0.64]; 0.76 | 60% (15.02, 0.024*) | NA |
| Intensity Matching IHT *vs.* NT  (Q = 1.15*,* *p =* 0.760) |  |  |  |  |
| % Same Relative Workload | 6 | -0.1 [-1.01, 0.81]; 0.791 | 69% (16.01, 0.007*) | NA |
| % of Normoxic Workload | 3 | 0.01 [-2.72; 2.74]; 0.816 | 65% (5.66, 0.060) | NA |
| % Same Relative HR_max_, or V̇O_2max_ | 8 | 0.25 [0.08, 0.42]; 0.009* | 0% (1.11, 0.993) | NA |
| % of Normoxic HR_max_, or V̇O_2max_ | 4 | 0.18 [-0.34, 0.7]; 0.352 | 0% (1.38, 0.710) | NA |
|  |  |  |  |  |
| **V̇O_2max_ Testing** |  |  |  |  |
| Gas Sampling (Q = 0.97*,* *p =* 0.610) |  |  |  |  |
| Breath-by-Breath | 16 | 0.14 [-0.16, 0.44]; 0.347 | 24% (19.74, 0.182) | 0.14 [-0.16, 0.44] |
| Douglas Bag | 3 | -0.13 [-2.61, 2.35]; 0.838 | 61% (5.15, 0.076) | NA |
| V̇O_2_ plateau criteria  (Q = 0.00*,* *p =* 0.980) |  |  |  |  |
| Yes | 10 | 0.13 [-0.26, 0.52]; 0.461 | 13% (10.29, 0.327) | 0.13 [-0.26, 0.52] |
| Not Specified | 11 | 0.13 [-0.28, 0.54]; 0.508 | 32% (14.61, 0.147) | 0.13 [-0.28, 0.54] |
| RER value criteria  (Q = 4.03*,* *p =* 0.130) |  |  |  |  |
| 1.0 | 6 | 0.12 [-0.2, 0.44]; 0.389 | 0% (1.56, 0.906) | NA |
| 1.1 | 3 | -0.64 [-3.11, 1.84]; 0.382 | 76% (8.41, 0.015*) | NA |
| Not Specified | 12 | 0.34 [0.09, 0.58]; 0.011* | 0% (7.24, 0.78) | 0.44 [0.18, 0.71] |
| La_max_ ≥ 8 mmol∙L^-1^ criteria  (Q = 0.00*,* *p =* 0.990) |  |  |  |  |
| Yes | 13 | 0.14 [-0.23, 0.5]; 0.428 | 21% (15.25, 0.228) | 0.14 [-0.23, 0.5] |
| Not Specified | 8 | 0.13 [-0.33, 0.59]; 0.521 | 28% (9.69, 0.207) | NA |
| Encouragement (Q = 0.05*,* *p =* 0.820) |  |  |  |  |
|  |  |  |  |  |
| Not Specified | 19 | 0.13 [-0.15, 0.42]; 0.337 | 28% (24.93, 0.127) | 0.13 [-0.15, 0.42] |
|  |  |  |  |  |
| **Publication Characteristics** |  |  |  |  |
| Randomization (Q = 0.13*,* *p =* 0.720) |  |  |  |  |
| None, or Not Specified | 6 | 0.06 [-0.51, 0.63]; 0.794 | 0% (4.41, 0.492) | NA |
| Yes | 15 | 0.16 [-0.16, 0.48]; 0.31 | 31% (20.31, 0.121) | 0.16 [-0.16, 0.48] |
| Blind (Q = 2.39*,* *p =* 0.120) |  |  |  |  |
| None, or Not Specified | 19 | 0.1 [-0.19, 0.38]; 0.485 | 25% (24.05, 0.153) | 0.1 [-0.19, 0.38] |
| Decade of Publication  (Q = 1.96*,* *p =* 0.370) |  |  |  |  |
| Before 2000 | 4 | -0.08 [-1.25, 1.1]; 0.849 | 25% (3.99, 0.263) | NA |
| 2000 - 2010 | 7 | -0.03 [-0.57, 0.5]; 0.887 | 27% (8.27, 0.219) | NA |
| After 2010 | 10 | 0.30 [-0.06, 0.66]; 0.093 | 14% (10.42, 0.318) | 0.30 [-0.06, 0.66] |
| Open Access Publication  (Q = 1.87*,* *p =* 0.170) |  |  |  |  |
| No | 12 | -0.02 [-0.38, 0.34]; 0.916 | 11% (12.29, 0.342) | -0.02 [-0.38, 0.34] |
| Yes | 9 | 0.31 [-0.1, 0.72]; 0.116 | 22% (10.26, 0.247) | NA |
|  |  |  |  |  |

**Supplementary Table 2.c.** Discrete Moderator Analysis for Peak Power Output (PPO)

|  | ***k*** | **SMD [95% CI]; *p-value*** | **I^2^ (Q, *p-value*)** | **Trim and Fill SMD [95% CI]** |
| --- | --- | --- | --- | --- |
| **Population Characteristics** |  |  |  |  |
| Sex (Q = 0.14*,* *p =* 0.710) |  |  |  |  |
| Male | 15 | 0.14 [-0.08, 0.36]; 0.206 | 0% (10.32, 0.739) | 0.21 [-0.01, 0.43] |
| Athletic Background  (Q = 3.27*,* *p =* 0.660) |  |  |  |  |
| Cyclists | 4 | 0.45 [-0.31, 1.22]; 0.156 | 0% (2.86, 0.415) | NA |
| Sedentary | 8 | 0.06 [-0.24, 0.35]; 0.668 | 0% (4.55, 0.715) | NA |
| Training Status (Q = 1.04*,* *p =* 0.310) |  |  |  |  |
| Untrained | 8 | 0.06 [-0.24, 0.35]; 0.668 | 0% (4.55, 0.715) | NA |
| Competitive | 8 | 0.26 [-0.1, 0.62]; 0.134 | 0% (5.18, 0.638) | NA |
| Country (Q = 43.87*,* p < 0.001*) |  |  |  |  |
| Asia (Other) | 3 | 0.34 [-0.14, 0.81]; 0.092 | 0% (0.43, 0.807) | NA |
| Europe (Other) | 4 | -0.17 [-0.45, 0.11]; 0.15 | 0% (0.46, 0.928) | NA |
| Poland | 3 | 0.71 [0.24, 1.17]; 0.022* | 0% (0.3, 0.863) | NA |
| Switzerland | 5 | -0.02 [-0.44, 0.41]; 0.923 | 0% (1.73, 0.784) | NA |
|  |  |  |  |  |
| **Training, Hypoxia Characteristics** |  |  |  |  |
| Training Schedule  (Q = 0.29*,* *p =* 0.590) |  |  |  |  |
| Exclusive | 11 | 0.11 [-0.14, 0.36]; 0.355 | 0% (6.77, 0.747) | 0.21 [-0.06, 0.47] |
| Integrated | 5 | 0.24 [-0.34, 0.81]; 0.317 | 0% (3.45, 0.486) | NA |
| Exercise Intensity  (Q = 0.52*,* *p =* 0.470) |  |  |  |  |
| High Intensity | 9 | 0.21 [-0.11, 0.53]; 0.171 | 0% (6.22, 0.622) | NA |
| Moderate Intensity | 7 | 0.07 [-0.28, 0.41]; 0.661 | 0% (3.86, 0.695) | NA |
| Method of Hypoxia  (Q = 1.07*,* *p =* 0.590) |  |  |  |  |
| Normobaric Hypoxic Chamber | 7 | 0.26 [-0.09, 0.6]; 0.123 | 0% (4.18, 0.653) | NA |
| Hypoxicator | 8 | 0.05 [-0.3, 0.4]; 0.752 | 0% (5.48, 0.602) | NA |
| Intensity Matching IHT *vs.* NT  (Q = 0.62*,* *p =* 0.890) |  |  |  |  |
| % Same Relative Workload | 6 | 0.12 [-0.34, 0.58]; 0.533 | 0% (4.82, 0.438) | NA |
| % of Normoxic Workload | 3 | 0.30 [-0.02, 1.63]; 0.618 | 15% (2.36, 0.310) | NA |
| % Same Relative HR_max_, or V̇O_2max_ | 5 | 0.07 [-0.4, 0.54]; 0.703 | 0% (2.52, 0.641) | NA |
|  |  |  |  |  |
| **V̇O_2max_ Testing** |  |  |  |  |
| Gas Sampling  (Q = 10.16*,* *p =* 0.001*) |  |  |  |  |
| Breath-by-Breath | 13 | 0.25 [0.02, 0.48]; 0.037* | 0% (7.51, 0.822) | 0.34 [0.1, 0.57] |
| Douglas Bag | 3 | -0.21 [-0.63, 0.21]; 0.164 | 0% (0.31, 0.857) | NA |
| V̇O_2_ plateau criteria  (Q = 0.00*,* *p >* 0.999) |  |  |  |  |
| Yes | 8 | 0.14 [-0.25, 0.54]; 0.419 | 0% (6.42, 0.492) | NA |
| Not Specified | 8 | 0.15 [-0.14, 0.43]; 0.263 | 0% (4.03, 0.776) | NA |
| RER value criteria  (Q = 5.98*,* *p =* 0.053) |  |  |  |  |
| 1.0 | 3 | 0.15 [-0.34, 0.64]; 0.311 | 0% (0.3, 0.863) | NA |
| 1.1 | 5 | -0.16 [-0.55, 0.22]; 0.306 | 0% (1.67, 0.796) | NA |
| Not Specified | 8 | 0.31 [-0.02, 0.63]; 0.063 | 0% (5.41, 0.61) | NA |
| La_max_ ≥ 8 mmol∙L^-1^ criteria  (Q = 2.12*,* *p =* 0.140) |  |  |  |  |
| Yes | 8 | 0.3 [-0.07, 0.67]; 0.098 | 0% (5.6, 0.588) | NA |
| Not Specified | 8 | 0.02 [-0.25, 0.28]; 0.874 | 0% (3.45, 0.84) | NA |
| Encouragement (Q = 0.87*,* *p =* 0.350) |  |  |  |  |
| Yes | 5 | 0.01 [-0.41, 0.43]; 0.962 | 0% (1.64, 0.802) | NA |
| Not Specified | 11 | 0.19 [-0.09, 0.46]; 0.156 | 0% (8.37, 0.593) | 0.19 [-0.09, 0.46] |
|  |  |  |  |  |
| **Publication Characteristics** |  |  |  |  |
| Randomization (Q = 0.23*,* *p =* 0.630) |  |  |  |  |
| None, or Not Specified | 3 | 0.07 [-0.48, 0.62]; 0.623 | 0% (0.34, 0.844) | NA |
| Yes | 13 | 0.16 [-0.1, 0.41]; 0.201 | 0% (10.05, 0.611) | 0.24 [-0.01, 0.5] |
| Blind (Q = 0.80*,* *p =* 0.370) |  |  |  |  |
| None, or Not Specified | 15 | 0.18 [-0.04, 0.4]; 0.101 | 0% (9.68, 0.786) | 0.3 [0.07, 0.54] |
| Decade of Publication  (Q = 5.60*,* *p =* 0.020*) |  |  |  |  |
| 2000 - 2010 | 10 | -0.03 [-0.24, 0.17]; 0.712 | 0% (3.2, 0.956) | -0.03 [-0.24, 0.17] |
| After 2010 | 6 | 0.40 [-0.01, 0.82]; 0.054 | 0% (3.87, 0.568) | NA |
| Open Access Publication  (Q = 7.71*,* *p =* 0.005*) |  |  |  |  |
| No | 11 | -0.04 [-0.23, 0.14]; 0.624 | 0% (3.24, 0.975) | -0.04 [-0.23, 0.14] |
| Yes | 5 | 0.47 [0.01, 0.92]; 0.046* | 0% (2.84, 0.586) | NA |
|  |  |  |  |  |

**Supplementary Table 2.d.** Discrete Moderator Analysis for Hemoglobin Concentration (Hb)

| **Population Characteristics** | ***k*** | **SMD [95% CI]; *p-value*** | **I^2^ (Q, *p-value*)** | **Trim and Fill SMD [95% CI]** |
| --- | --- | --- | --- | --- |
| Sex (Q = 0.29*,* *p =* 0.870) |  |  |  |  |
| Male | 14 | 0.11 [-0.08, 0.29]; 0.241 | 0% (6.1, 0.943) | 0.03 [-0.17, 0.23] |
| Athletic Background  (Q = 8.64*,* *p =* 0.120) |  |  |  |  |
| Cyclists | 3 | -0.06 [-1.05, 0.94]; 0.828 | 0% (1.36, 0.506) | NA |
| Runners | 3 | 0.18 [-0.06, 0.42]; 0.083 | 0% (0.08, 0.961) | NA |
| Sedentary | 5 | 0.28 [-0.08, 0.65]; 0.099 | 0% (1.86, 0.762) | NA |
| Training Status (Q = 2.52*,* *p =* 0.110) |  |  |  |  |
| Untrained | 5 | 0.28 [-0.08, 0.65]; 0.099 | 0% (1.86, 0.762) | NA |
| Competitive | 11 | 0.03 [-0.16, 0.23]; 0.724 | 0% (3.46, 0.969) | -0.03 [-0.22, 0.16] |
| Country (Q = 10.06*,* *p =* 0.070) |  |  |  |  |
| Asia (Other) | 3 | 0.16 [-0.28, 0.59]; 0.262 | 0% (0.37, 0.833) | NA |
| Europe (Other) | 3 | 0.3 [-1.03, 1.62]; 0.439 | 9% (2.19, 0.335) | NA |
| Poland | 4 | -0.02 [-0.59, 0.54]; 0.91 | 0% (1.56, 0.668) | NA |
|  |  |  |  |  |
| **Training, Hypoxia Characteristics** |  |  |  |  |
| Training Schedule  (Q = 1.52*,* *p =* 0.220) |  |  |  |  |
| Exclusive | 9 | 0.2 [-0.06, 0.45]; 0.11 | 0% (4.17, 0.842) | NA |
| Integrated | 7 | 0.02 [-0.22, 0.26]; 0.873 | 0% (1.64, 0.95) | NA |
| Exercise Intensity  (Q = 2.14*,* *p =* 0.140) |  |  |  |  |
| High Intensity | 11 | 0.04 [-0.16, 0.24]; 0.655 | 0% (3.6, 0.964) | -0.02 [-0.21, 0.17] |
| Moderate Intensity | 5 | 0.28 [-0.1, 0.65]; 0.109 | 0% (1.85, 0.763) | NA |
| Type of Exercise  (Q = 1.22*,* *p =* 0.540) |  |  |  |  |
| Cycling | 11 | 0.17 [-0.06, 0.39]; 0.13 | 0% (5.04, 0.889) | 0.08 [-0.15, 0.32] |
| Running | 4 | 0.15 [-0.04, 0.33]; 0.084 | 0% (0.16, 0.984) | NA |
| Method of Hypoxia  (Q = 0.81*,* *p =* 0.670) |  |  |  |  |
| Hypobaric Hypoxic Chamber | 3 | 0.03 [-0.84, 0.89]; 0.911 | 0% (1.07, 0.585) | NA |
| Normobaric Hypoxic Chamber | 8 | 0.12 [-0.19, 0.44]; 0.39 | 0% (4.74, 0.691) | NA |
| Hypoxicator | 5 | 0.2 [0.02, 0.37]; 0.036* | 0% (0.32, 0.988) | NA |
| Intensity Matching IHT *vs.* NT  (Q = 4.30*,* *p =* 0.230) |  |  |  |  |
| % Same Relative Workload | 6 | 0.09 [-0.31, 0.5]; 0.589 | 0% (3.73, 0.589) | NA |
| % of Normoxic Workload | 3 | 0.04 [-0.54; 0.62]; 0.828 | 0% (0.47, 0.790) | NA |
| % Same Relative HR_max_, or V̇O_2max_ | 4 | 0.14 [-0.46, 0.75]; 0.514 | 0% (1.71, 0.635) | NA |
| % of Normoxic HR_max_, or V̇O_2max_ | 3 | 0.28 [0.11, 0.45]; 0.020* | 0% (0.04, 0.98) | NA |
|  |  |  |  |  |
| **V̇O_2max_ Testing** |  |  |  |  |
| Gas Sampling (Q = 0.17*,* *p =* 0.680) |  |  |  |  |
| Breath-by-Breath | 13 | 0.09 [-0.07, 0.25]; 0.225 | 0% (3.8, 0.987) | 0.06 [-0.1, 0.21] |
| V̇O_2_ plateau criteria  (Q = 7.16*,* *p =* 0.007*) |  |  |  |  |
| Yes | 8 | 0.31 [0.1, 0.51]; 0.010* | 0% (1.75, 0.972) | NA |
| Not Specified | 7 | -0.06 [-0.32, 0.2]; 0.593 | 0% (2.3, 0.89) | NA |
| RER value criteria  (Q = 5.72*,* *p =* 0.060) |  |  |  |  |
| 1.0 | 5 | 0.2 [0.01, 0.38]; 0.040* | 0% (0.32, 0.988) | NA |
| 1.1 | 3 | 0.44 [-0.36, 1.23]; 0.142 | 0% (0.91, 0.635) | NA |
| Not Specified | 7 | -0.04 [-0.32, 0.24]; 0.722 | 0% (2.72, 0.843) | NA |
| La_max_ ≥ 8 mmol∙L^-1^ criteria  (Q = 0.04*,* *p =* 0.840) |  |  |  |  |
| Yes | 8 | 0.14 [-0.1, 0.37]; 0.22 | 0% (2.41, 0.934) | NA |
| Not Specified | 7 | 0.1 [-0.24, 0.44]; 0.488 | 0% (3.89, 0.692) | NA |
| Encouragement (Q = 0.03*,* *p =* 0.860) |  |  |  |  |
| Yes | 5 | 0.14 [-0.15, 0.44]; 0.242 | 0% (0.86, 0.931) | NA |
| Not Specified | 11 | 0.12 [-0.11, 0.35]; 0.277 | 0% (5.5, 0.855) | 0.03 [-0.22, 0.27] |
|  |  |  |  |  |
| **Publication Characteristics** |  |  |  |  |
| Randomization (Q = 1.11*,* *p =* 0.290) |  |  |  |  |
| None, or Not Specified | 4 | -0.04 [-0.6, 0.52]; 0.85 | 0% (1.41, 0.702) | NA |
| Yes | 12 | 0.17 [-0.02, 0.36]; 0.072 | 0% (4.45, 0.955) | 0.07 [-0.12, 0.27] |
| Blind (Q = 0.06*,* *p =* 0.810) |  |  |  |  |
| None, or Not Specified | 15 | 0.12 [-0.06, 0.29]; 0.167 | 0% (6.32, 0.958) | 0.05 [-0.13, 0.24] |
| Decade of Publication  (Q = 3.55*,* *p =* 0.060) |  |  |  |  |
| 2000 - 2010 | 8 | 0.28 [0.03, 0.53]; 0.034* | 0% (2.45, 0.931) | NA |
| After 2010 | 8 | 0.01 [-0.22, 0.24]; 0.945 | 0% (2.64, 0.916) | NA |
| Open Access Publication  (Q = 4.26*,* *p =* 0.040*) |  |  |  |  |
| No | 9 | 0.27 [0.06, 0.49]; 0.019* | 0% (2.45, 0.964) | NA |
| Yes | 7 | -0.02 [-0.27, 0.24]; 0.881 | 0% (2.42, 0.878) | NA |
|  |  |  |  |  |

**Supplementary Table 2.e.** Discrete Moderator Analysis for Hematocrit (Ht)

|  | ***k*** | **SMD [95% CI]; *p-value*** | **I^2^ (Q, *p-value*)** | **Trim and Fill SMD [95% CI]** |
| --- | --- | --- | --- | --- |
| **Population Characteristics** |  |  |  |  |
| Sex (Q = 2.92*,* *p =* 0.230) |  |  |  |  |
| Male | 13 | 0.01 [-0.21, 0.24]; 0.894 | 0% (7.54, 0.82) | -0.19 [-0.43, 0.05] |
| Athletic Background  (Q = 7.36*,* *p =* 0.200) |  |  |  |  |
| Cyclists | 3 | 0.14 [-0.5, 0.77]; 0.454 | 0% (0.56, 0.757) | NA |
| Runners | 3 | 0.43 [-0.52, 1.37]; 0.191 | 0% (1.2, 0.548) | NA |
| Sedentary | 4 | 0.12 [-0.72, 0.96]; 0.683 | 0% (4.91, 0.179) | NA |
| Training Status (Q = 0.00*,* *p =* 0.960) |  |  |  |  |
| Untrained | 4 | 0.12 [-0.72, 0.96]; 0.683 | 0% (4.91, 0.179) | NA |
| Competitive | 11 | 0.1 [-0.14, 0.35]; 0.369 | 0% (5.39, 0.864) | 0.1 [-0.14, 0.35] |
| Country (Q = 0.72*,* *p =* 0.980) |  |  |  |  |
| Asia (Other) | 3 | 0.1 [-1.43, 1.63]; 0.811 | 1% (4.45, 0.108) | NA |
| Europe (Other) | 3 | 0.1 [-0.42, 0.62]; 0.509 | 0% (0.35, 0.839) | NA |
| Poland | 4 | 0.08 [-0.26, 0.43]; 0.489 | 0% (0.58, 0.902) | NA |
|  |  |  |  |  |
| **Training, Hypoxia Characteristics** |  |  |  |  |
| Training Schedule  (Q = 0.84*,* *p =* 0.360) |  |  |  |  |
| Exclusive | 8 | 0.17 [-0.23, 0.57]; 0.345 | 0% (7.77, 0.354) | NA |
| Integrated | 7 | -0.02 [-0.29, 0.26]; 0.897 | 0% (2.15, 0.905) | NA |
| Exercise Intensity  (Q = 0.03*,* *p =* 0.850) |  |  |  |  |
| High Intensity | 10 | 0.11 [-0.16, 0.39]; 0.375 | 0% (5.35, 0.803) | 0.11 [-0.16, 0.39] |
| Moderate Intensity | 5 | 0.07 [-0.53, 0.67]; 0.773 | 0% (4.91, 0.297) | NA |
| Type of Exercise  (Q = 3.62*,* *p =* 0.160) |  |  |  |  |
| Cycling | 10 | 0.06 [-0.2, 0.33]; 0.62 | 0% (5.66, 0.773) | -0.19 [-0.47, 0.09] |
| Running | 4 | 0.33 [-0.28, 0.95]; 0.184 | 0% (1.76, 0.624) | NA |
| Method of Hypoxia  (Q = 2.76*,* *p =* 0.250) |  |  |  |  |
| Hypobaric Hypoxic Chamber | 3 | 0.09 [-1.67, 1.84]; 0.852 | 1% (4.43, 0.109) | NA |
| Normobaric Hypoxic Chamber | 8 | 0.01 [-0.32, 0.34]; 0.964 | 0% (5.11, 0.646) | NA |
| Hypoxicator | 4 | 0.25 [0.07, 0.43]; 0.021* | 0% (0.15, 0.985) | NA |
| Intensity Matching IHT *vs.* NT  (Q = 5.85*,* *p =* 0.120) |  |  |  |  |
| % Same Relative Workload | 6 | -0.08 [-0.45, 0.29]; 0.589 | 0% (3.11, 0.683) | NA |
| % of Normoxic Workload | 3 | 0.27 [-0.46; 0.99]; 0.762 | 0% (0.72, 0.730) | NA |
| % Same Relative HR_max_, or V̇O_2max_ | 3 | -0.12 [-1.26, 1.03]; 0.703 | 0% (1.61, 0.448) | NA |
| % of Normoxic HR_max_, or V̇O_2max_ | 3 | 0.48 [-0.54, 1.5]; 0.179 | 0% (1.33, 0.513) | NA |
|  |  |  |  |  |
| **V̇O_2max_ Testing** |  |  |  |  |
| Gas Sampling (Q = 0.13*,* *p =* 0.720) |  |  |  |  |
| Breath-by-Breath | 12 | 0.05 [-0.23, 0.34]; 0.678 | 0% (9.78, 0.551) | -0.2 [-0.5, 0.11] |
| V̇O_2_ plateau criteria  (Q = 15.22*,* p < 0.001*) |  |  |  |  |
| Yes | 7 | 0.4 [0.14, 0.65]; 0.009* | 0% (1.81, 0.936) | NA |
| Not Specified | 7 | -0.21 [-0.49, 0.07]; 0.122 | 0% (2.7, 0.845) | NA |
| RER value criteria  (Q = 8.06*,* *p =* 0.020*0) |  |  |  |  |
| 1.0 | 5 | 0.23 [-0.12, 0.58]; 0.137 | 0% (1.17, 0.882) | NA |
| Not Specified | 7 | -0.15 [-0.49, 0.18]; 0.304 | 0% (3.9, 0.69) | NA |
| La_max_ ≥ 8 mmol∙L^-1^ criteria  (Q = 6.13*,* *p =* 0.011*) |  |  |  |  |
| Yes | 7 | 0.32 [0, 0.64]; 0.051 | 0% (2.95, 0.815) | NA |
| Not Specified | 7 | -0.15 [-0.48, 0.18]; 0.314 | 0% (3.77, 0.707) | NA |
| Encouragement (Q = 0.25*,* *p =* 0.620) |  |  |  |  |
| Yes | 4 | 0.14 [-0.14, 0.42]; 0.202 | 0% (0.35, 0.951) | NA |
| Not Specified | 11 | 0.06 [-0.25, 0.37]; 0.668 | 0% (9.95, 0.445) | -0.16 [-0.5, 0.17] |
|  |  |  |  |  |
| **Publication Characteristics** |  |  |  |  |
| Randomization (Q = 1.80*,* *p =* 0.180) |  |  |  |  |
| None, or Not Specified | 4 | -0.14 [-0.7, 0.41]; 0.475 | 0% (1.38, 0.709) | NA |
| Yes | 11 | 0.15 [-0.13, 0.43]; 0.271 | 0% (8.02, 0.627) | -0.03 [-0.32, 0.25] |
| Blind (Q = 0.24*,* *p =* 0.620) |  |  |  |  |
| None, or Not Specified | 14 | 0.07 [-0.18, 0.31]; 0.566 | 0% (10.13, 0.683) | -0.18 [-0.45, 0.08] |
| Decade of Publication  (Q = 1.72*,* *p =* 0.190) |  |  |  |  |
| 2000 - 2010 | 7 | 0.24 [0.02, 0.46]; 0.039* | 0% (1.31, 0.971) | NA |
| After 2010 | 8 | -0.01 [-0.42, 0.39]; 0.935 | 0% (7.92, 0.34) | NA |
| Open Access Publication  (Q = 2.09*,* *p =* 0.150) |  |  |  |  |
| No | 8 | 0.25 [0.06, 0.44]; 0.016* | 0% (1.34, 0.987) | NA |
| Yes | 7 | -0.04 [-0.5, 0.41]; 0.822 | 0% (7.41, 0.284) | NA |
|  |  |  |  |  |

**Supplementary Table 2.f.** Independent Group Analysis for Time to Exhaustion (TTE)

|  | ***k*** | **SMD [95% CI]; *p-value*** | **I^2^ (Q, *p-value*)** | **Trim and Fill**  **SMD [95% CI]** |
| --- | --- | --- | --- | --- |
| **Population Characteristics** |  |  |  |  |
| Athletic Background (NA) |  |  |  |  |
| Sedentary | 4 | -0.51 [-0.49, 0.48]; 0.200 | 8% (3.27, 0.351) | NA |
|  |  |  |  |  |
| **Training, Hypoxia Characteristics** |  |  |  |  |
| Exercise Intensity (NA) |  |  |  |  |
| Moderate Intensity | 4 | -0.51 [-0.49, 0.48]; 0.200 | 8% (3.27, 0.351) | NA |
| Type of Exercise (NA) |  |  |  |  |
| Cycling | 4 | -0.51 [-0.49, 0.48]; 0.200 | 8% (3.27, 0.351) | NA |
| Method of Hypoxia (NA) |  |  |  |  |
| Hypobaric Hypoxic Chamber | 3 | -0.43 [-2.14, 1.29]; 0.396 | 26% (2.71, 0.258) | NA |
|  |  |  |  |  |
| **V̇O_2max_** **Testing** |  |  |  |  |
| V̇O_2_ plateau criteria (NA) |  |  |  |  |
| Not Specified | 4 | -0.51 [-0.49, 0.48]; 0.200 | 8% (3.27, 0.351) | NA |
| RER value criteria (NA) |  |  |  |  |
| Not Specified | 4 | -0.51 [-0.49, 0.48]; 0.200 | 8% (3.27, 0.351) | NA |
| La_max_ ≥ 8 mmol∙L^-1^ criteria (NA) |  |  |  |  |
| Yes | 4 | -0.51 [-0.49, 0.48]; 0.200 | 8% (3.27, 0.351) | NA |
| Encouragement (NA) |  |  |  |  |
| Not Specified | 4 | -0.51 [-0.49, 0.48]; 0.200 | 8% (3.27, 0.351) | NA |
|  |  |  |  |  |
| **Publication Characteristics** |  |  |  |  |
| Randomization (NA) |  |  |  |  |
| Yes | 3 | 0.00 [-1.79, 1.79]; 0.997 | 41% (3.40, 0.183) | NA |
| Blind (NA) |  |  |  |  |
| None, or Not Specified | 3 | -0.93 [-1.1, -0.76]; 0.002* | 0% (0.02, 0.99) | NA |
| Decade of Publication (NA) |  |  |  |  |
| Before 2000 | 3 | -0.43 [-2.14, 1.29]; 0.396 | 26% (2.71, 0.258) | NA |
|  |  |  |  |  |
|  |  |  |  |  |

CI: Confidence Interval; HR_max_: Maximum Heart Rate; k: Number of reports included in the subgroup; Laₘₐₓ: Maximum blood lactate concentration; NA: Not Applicable; Q: Cochran’s Q test; RER: Respiratory Exchange Ratio; SMD: Standardized Mean Difference; V̇O_2max_: Maximal Oxygen Uptake
