# Supplementary material for: Aerobic Intermittent Hypoxic Training Is Not Beneficial for Maximal Oxygen Uptake and Performance: A Systematic Review and Meta‐Analysis
Source: Scand J Med Sci Sports. 2025 Jun 23;35(6):e70088. doi: 10.1111/sms.70088 (PMC12184621; doi:10.1111/sms.70088)
Supplement: Supplementary file 5 — Table S3: [file SMS-35-e70088-s001.docx]

**Supplementary Table 3.** Meta-regression Analysis Results

|  | **β** | **Lower CI Value** | **Upper CI Value** | ***p*-value** |
| --- | --- | --- | --- | --- |
| **Maximal Oxygen Uptake (V̇O_2max_)** |  |  |  |  |
| Hypoxic Level |  |  |  |  |
| Fraction of Inspired Oxygen (%) | 3.E-02 | -1.E-01 | 2.E-01 | 0.659 |
| Simulated Altitude (meters above sea level) | -9.E-05 | -4.E-04 | 2.E-04 | 0.539 |
|  |  |  |  |  |
| Training Duration |  |  |  |  |
| Duration of Training (weeks) | 7.E-02 | -2.E-01 | 4.E-01 | 0.639 |
| Days of IHT | 6.E-03 | -5.E-02 | 6.E-02 | 0.843 |
| Total Volume of the Training (min) | -2.E-05 | -1.E-04 | 1.E-04 | 0.723 |
| Frequency |  |  |  |  |
| Days per week | -2.E-02 | -2.E-01 | 2.E-01 | 0.815 |
| Minutes per week | -7.E-05 | -4.E-04 | 3.E-04 | 0.711 |
| Minutes per day | -5.E-04 | -3.E-03 | 2.E-03 | 0.711 |
| Year of Publication | 1.E-03 | -2.E-02 | 2.E-02 | 0.920 |
|  |  |  |  |  |
| **Absolute Maximal Oxygen Uptake (_abs_V̇O_2max_)** | |  |  |  |
|  |  |  |  |  |
| Hypoxic Level |  |  |  |  |
| Fraction of Inspired Oxygen (%) | 9.E-01 | -4.E+00 | 5.E+00 | 0.701 |
| Simulated Altitude (meters above sea level) | -2.E-03 | -1.E-02 | 6.E-03 | 0.626 |
| Training Duration |  |  |  |  |
| Duration of Training (weeks) | -6.E-01 | -2.E+00 | 5.E-01 | 0.322 |
| Days of IHT | -8.E-02 | -2.E-01 | 4.E-02 | 0.170 |
| Total Volume of the Training (min) | 3.E-04 | -9.E-04 | 2.E-03 | 0.614 |
| Frequency |  |  |  |  |
| Days per week | -4.E-01 | -1.E+00 | 3.E-01 | 0.279 |
| Minutes per week | 8.E-04 | -2.E-03 | 4.E-03 | 0.596 |
| Minutes per day | 6.E-03 | -2.E-02 | 3.E-02 | 0.596 |
| Year of Publication | 7.E-02 | -2.E-02 | 1.E-01 | 0.118 |
|  |  |  |  |  |
| **Peak Power Output (PPO)** |  |  |  |  |
| Hypoxic Level |  |  |  |  |
| Fraction of Inspired Oxygen (%) | 2.E+00 | -1.E+00 | 6.E+00 | 0.197 |
| Simulated Altitude (meters above sea level) | -4.E-03 | -1.E-02 | 3.E-03 | 0.224 |
| Training Duration |  |  |  |  |
| Duration of Training (weeks) | -4.E-01 | -4.E+00 | 3.E+00 | 0.781 |
| Days of IHT | -1.E-01 | -6.E-01 | 3.E-01 | 0.626 |
| Total Volume of the Training (min) | -5.E-04 | -4.E-03 | 3.E-03 | 0.773 |
| Frequency |  |  |  |  |
| Days per week | -1.E+00 | -5.E+00 | 2.E+00 | 0.513 |
| Minutes per week | -1.E-03 | -1.E-02 | 8.E-03 | 0.825 |
| Minutes per day | -7.E-03 | -7.E-02 | 6.E-02 | 0.825 |
| Year of Publication | 5.E-01 | -5.E-02 | 1.E+00 | 0.076 |
|  |  |  |  |  |
| **Hemoglobin Concentration** |  |  |  |  |
| Hypoxic Level |  |  |  |  |
| Fraction of Inspired Oxygen (%) | -2.E-01 | -5.E-01 | 2.E-01 | 0.321 |
| Simulated Altitude (meters above sea level) | 4.E-04 | -3.E-04 | 1.E-03 | 0.300 |
| Training Duration |  |  |  |  |
| Duration of Training (weeks) | 3.E-02 | -2.E-01 | 3.E-01 | 0.811 |
| Days of IHT | 5.E-02 | -5.E-02 | 2.E-01 | 0.355 |
| Total Volume of the Training (min) | -1.E-04 | -3.E-04 | 8.E-05 | 0.277 |
| Frequency |  |  |  |  |
| Days per week | 3.E-02 | -1.E-01 | 1.E-01 | 0.680 |
| Minutes per week | -3.E-04 | -9.E-04 | 2.E-04 | 0.241 |
| Minutes per day | -2.E-03 | -6.E-03 | 2.E-03 | 0.241 |
| Year of Publication | -9.E-03 | -4.E-02 | 2.E-02 | 0.609 |
|  |  |  |  |  |
| **Hematocrit** |  |  |  |  |
| Hypoxic Level |  |  |  |  |
| Fraction of Inspired Oxygen (%) | -3.E-01 | -8.E-01 | 3.E-01 | 0.326 |
| Simulated Altitude (meters above sea level) | 5.E-04 | -5.E-04 | 1.E-03 | 0.318 |
| Training Duration |  |  |  |  |
| Duration of The Training (weeks) | -5.E-02 | -3.E-01 | 2.E-01 | 0.699 |
| Days of IHT | -3.E-02 | -9.E-02 | 4.E-02 | 0.453 |
| Total Volume of the Training (min) | -4.E-05 | -4.E-04 | 3.E-04 | 0.811 |
| Frequency |  |  |  |  |
| Days per week | -4.E-02 | -3.E-01 | 2.E-01 | 0.786 |
| Minutes per week | -8.E-05 | -1.E-03 | 1.E-03 | 0.879 |
| Minutes per day | -6.E-04 | -8.E-03 | 7.E-03 | 0.879 |
| Year of Publication | -2.E-02 | -9.E-02 | 5.E-02 | 0.514 |
|  |  |  |  |  |

**CI: 95% Confidence Interval; β**: regression coefficient; IHT: Intermittent Hypoxic Training
